# Judging facts, judging norms: Training machine learning models to judge humans requires a modified approach to labeling data

**DOI:** 10.1126/sciadv.abq0701

**Published:** 2023-05-10

**Authors:** Aparna Balagopalan, David Madras, David H. Yang, Dylan Hadfield-Menell, Gillian K. Hadfield, Marzyeh Ghassemi

**Affiliations:** ^1^ Massachusetts Institute of Technology, Cambridge, MA, USA.; ^2^ University of Toronto, Toronto, Ontario, Canada.; ^3^ Vector Institute, Toronto, Ontario, Canada.; ^4^ ML Estimation, Toronto, Ontario, Canada.; ^5^ Schwartz Reisman Institute for Technology and Society, Toronto, Ontario, Canada.; ^6^ Center for Human-Compatible AI, Berkeley, CA, USA.; ^7^ OpenAI, San Francisco, CA, USA.

## Abstract

As governments and industry turn to increased use of automated decision systems, it becomes essential to consider how closely such systems can reproduce human judgment. We identify a core potential failure, finding that annotators label objects differently depending on whether they are being asked a factual question or a normative question. This challenges a natural assumption maintained in many standard machine-learning (ML) data acquisition procedures: that there is no difference between predicting the factual classification of an object and an exercise of judgment about whether an object violates a rule premised on those facts. We find that using factual labels to train models intended for normative judgments introduces a notable measurement error. We show that models trained using factual labels yield significantly different judgments than those trained using normative labels and that the impact of this effect on model performance can exceed that of other factors (e.g., dataset size) that routinely attract attention from ML researchers and practitioners.

## Teaser

Machine learning systems trained with factual features as labels do not reproduce human rule violation judgments on the same data.

## INTRODUCTION

Building systems to make or support normative judgments is an important goal and use case for machine learning (ML). Such systems, if designed well to accurately implement human rules and norms, hold the promise of reducing backlogs and decision-making costs (*1*), improving fairness [by eliminating judgment failures such as the failure to ignore irrelevant facts (*2*)], and increasing access to neutral adjudication (*3*). Existing use cases include automated decision-making in employment (*4*), credit risk assessment (*5*), criminal justice (*3*), and government services (*6*) contexts. Governments around the world are beginning to regulate the use of automated decision-making in both the public and private sectors (*7*, *8*).

Human systems of rules and norms, including legal systems, consist of factual predicates to which normative judgment is applied. If someone thinks that a legal rule has been violated, they allege that the factual predicates of the legal rule exist, warranting a legal judgment of violation [note that the distinction between a fact and a legal judgment is complex in legal reasoning; for a discussion, see (*9*)]. In a well-functioning legal regime, decisions exercising normative judgment are supported by justification (*10*): an appeal to the reasons justifying the decision by showing that it is consistent with the rules intended to govern the decision. For example, if a post displayed on a social media platform is judged as a violation of the platform’s code of conduct, a system should be able to justify that conclusion using a valid reason, e.g., the post included dehumanizing speech about members of a particular religious group or ethnicity (see, for example, community standards for hate speech on social media platforms at https://transparency.fb.com/policies/community-standards/hate-speech/), and the code prohibits such comments. Factual determinations may be subjective and involve disagreements: e.g., there is often no “ground truth” on what constitutes dehumanizing speech (*11*). However, legal systems use procedural rules (such as requiring a unanimous jury) to resolve these disagreements, either to make factual findings or to render judgments on the basis of factual findings.

A seemingly reasonable approach to scale these procedures is to train classifiers on the presence of the factual predicates (factual features of a given data point) of a normative rule. For example, to detect violations of a social media platform’s code of conduct at scale, one might build a model to classify the presence of the factual features prohibited by the code, such as dehumanizing speech about a religious group or ethnicity, and then apply an if-then logic to transform factual classification into normative judgments: if dehumanizing speech, then violation.

Here, we demonstrate a flaw in this seemingly reasonable approach: It fails to faithfully reproduce human judgment about norm violations. We conduct human subject experiments showing that humans judge the abstract presence of factual features significantly differently than they judge norm violations, even when norm violations depend entirely on the presence of factual features. Moreover, there is considerable variance in both factual and normative judgments, but variance in factual judgments does not translate neatly into variance in normative judgment. We report findings in four contexts—using images of clothing, meals, and pets and text snippets from a discussion forum—and demonstrate that humans are systematically less likely to say that a rule has been violated than to say that the relevant factual features (on which that same rule is predicated) are present.

Next, we consider the impact of this difference on ML models. We construct two datasets with the same objects but different labels: a descriptive set of labels, where we asked labelers to identify factual features of an image or text, and we then applied a rule to indirectly determine violation, and a normative set of labels, where we asked labelers to directly judge whether an image or text violates a rule based on those factual features. We train supervised ML models [ResNet-50 (*12*) and ALBERT (*13*)] on both datasets. We find that the differences in the data are reflected in the trained models. A model trained with descriptive labels exhibits significantly poorer performance in judging violations (i.e., predicting human-annotated normative judgments) in comparison to one trained with normative labels. Specifically, we find that models trained using descriptive labels are more likely to judge rules to be violated than human judges in all four settings.

Last, we demonstrate that this model performance gap is comparable to, or greater than, the performance gap incurred between various other model architecture and data choices that are the frequent focus of efforts to improve model performance (e.g., label noise or dataset size). This performance gap between descriptive and normative labeling is a critical insight, specifically because data for ML systems are often collected in a descriptive setting (*11*, *14*–*16*). Our findings demonstrate that ML models intended to augment or automate human normative judgments will routinely make sizable errors if trained on descriptive labels. In our specific cases, this means that models routinely overpredict rule violations.

**Fig. 1.**
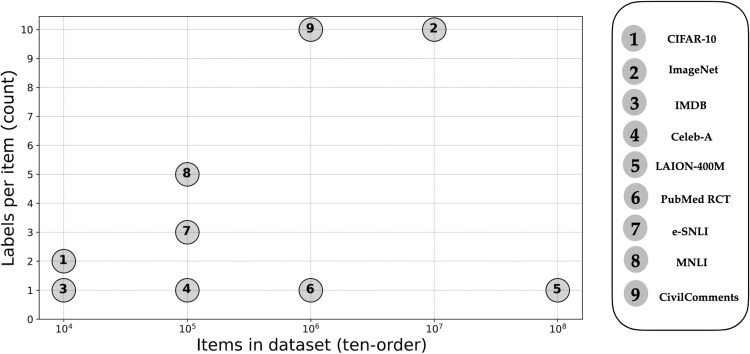
Large ML datasets are increasingly important, but multiple data labels per item are rarely available. Figure shows size versus the number of labels per item for some popular datasets (*14*, *15*, *30*, *58*, *60*–*64*). Reference numbers are (*60*, *14*, *61*, *15*, *62*, *63*, *64*, *30*, *58*) in order corresponding to datasets indicated as points 1 to 9, respectively, in the figure.

Our study identifies an important phenomenon about how humans exercise judgment and normative reasoning (*17*) that is unaccounted for in ML. Our analysis has deep implications for how we build, collect data for, and evaluate ML systems that are intended to operate in normative settings. Current ML training practices pay little attention to data labeling as a matter of complex human behavior (*11*, *18*). Many large widely used datasets collect only a handful of labels per item (see Fig. 1), failing to recognize the potential for variance in human perception of objects, even on factual criteria. Our study highlights the importance of collecting multiple labels per object to assess the labeling variability across participating labelers. As for the training of automated decision systems to exercise normative judgment, there is little voluntary disclosure of the specific procedures used to collect labels, which we identify as critical. While some systems have been trained using historical normative decisions (not collected explicitly for model development) as targets for training models (*3*, *5*), the dearth of information makes it difficult to prove what we strongly suspect is true: that it is standard practice to train systems for normative applications on the basis of factual labels alone and routinely on labels acquired for a different purpose that can be acquired inexpensively from publicly available sources. Concrete examples we could identify include using factual labels to construct normative judgments for violations of a dress code for a construction site (*19*), rules prohibiting toxic content online (e.g., Perspective API; https://perspectiveapi.com), and proctoring rules for online test taking (*20*). We expand on these observations in Discussion.

Our results suggest that a critical consideration has been overlooked in these cases: Normative judgment is a holistic process that cannot be neatly decomposed into factual prediction and rule application. Approaches to automation based on this decomposition may create a false promise of objectivity. Full appreciation of this consideration would require more careful, and expensive, efforts to curate data expressly for the normative context in which they are to be deployed. The growing literature on weaknesses in the capacity of humans to make appropriate use of ML predictions (*21*, *22*), even in expert settings (*23*), underscores the importance of paying close attention to the complex nature of human-machine interaction when it comes to exercising judgment, and our results lend a new dimension to this urgent problem.

## RESULTS

### Data acquisition in normative and descriptive settings

**Fig. 2.**
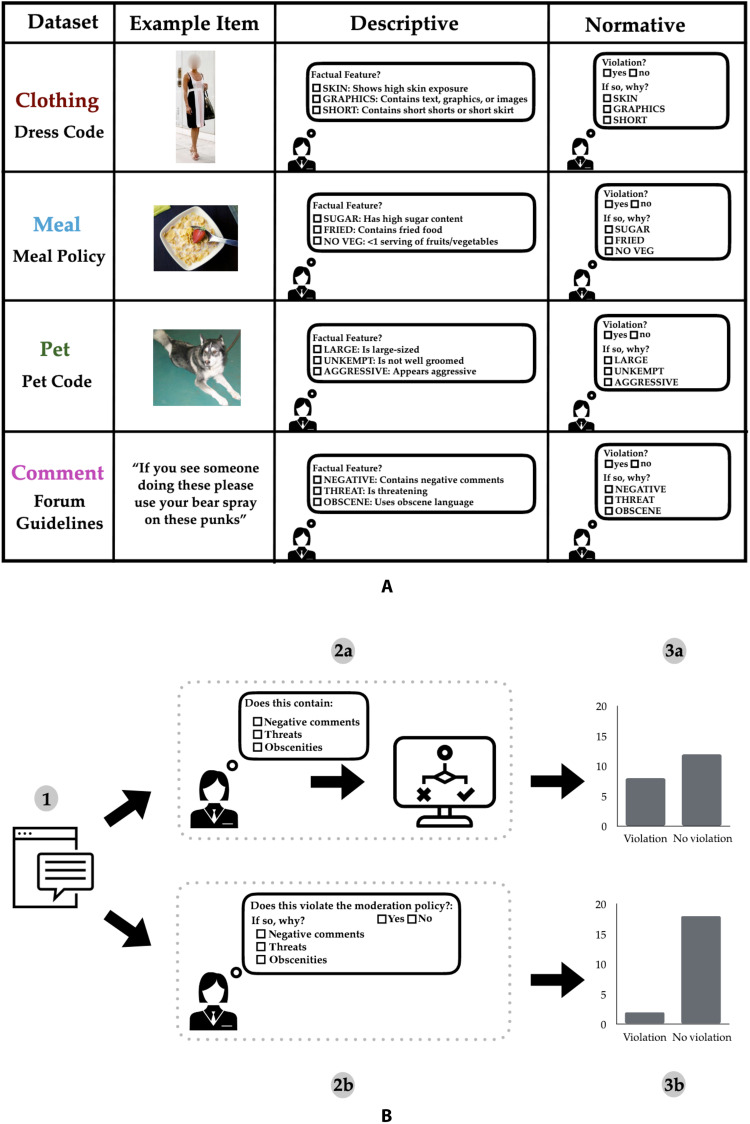
We contrast rule violation judgment labels collected under a normative condition with those constructed using factual feature labels collected under a descriptive condition. (**A**) We collect descriptive labels, (i.e., involving assessments of factual features) and normative rule violation judgment labels for four settings: Clothing (a dress code), Meal (a school meal policy), Pet (a building pet code), and Comment (an online discussion forum’s comment policy). (**B**) Contrasting data collection prompts asking labelers to make descriptive judgments (A), instead of directly assessing rule violations normatively (B). Note that example image and text objects shown in the figure are from open-sourced, publicly available datasets.

We acquired normative and descriptive labels in four stylized settings to test for differences in how humans judge facts and norms. Three of these settings concerned judgments about images, and the fourth about short text samples. We constructed fictional rules (or codes) governing four settings: Clothing mimics a dress code for clothing worn in an office or school setting, Meal mimics a policy for meals served in schools, Pet mimics a pet code for dogs permitted in apartment buildings, and Comment mimics guidelines for comments posted in online forums (Fig. 2A).

We aimed to keep our constructed codes realistic but simple, using only three factual features for each code. Further, we ensured that factual features do not depend on judging mental states such as a person’s intent or require additional specialist knowledge. For example, we included “high sugar content” in the Meal setting rather than query about potential allergens, and we excluded features such as “sexually provocative” from our dress code. This was done to emulate settings where a model might plausibly succeed in classifying factual features. Note, however, that we did include factual features with elements of subjectivity, and for which we could expect variance in labeling (e.g., what size does a dog have to be to be labeled “large”?).

For each setting, we presented participants recruited through Amazon’s Mechanical Turk (MT) with an object (image or text sample) and asked them to answer questions (Fig. 2B). We allocated participants in each setting to one of two treatment conditions:

1) Descriptive condition: Participants were presented with three factual features included in a relevant rule and asked to indicate (yes/no) whether each was present in the object. Participants were not given the relevant rule nor was the task framed in the context of ultimately judging rule violations. Responses were then used to construct normative judgments for the object using if-then logic: If a participant indicated that any of the three features of the relevant rule were present, then the object was deemed to be in violation of the rule for that participant.

2) Normative condition: Participants were given the relevant rule and asked whether (yes/no) the rule was violated—that is, normative judgments were elicited. Participants were then asked to identify all the reasons that justified their judgment by selecting which of the factual features included in the relevant rule were present.

Each setting—referred to henceforth as a dataset—contained 2000 objects, each labeled by 20 participants in both conditions: a total of 40,000 data points in each condition. All objects in each case were sampled from existing datasets (details in Supplementary Text). We refer to the judgment labels generated by applying if-then logic to the feature labels collected in the descriptive condition as descriptive labels and those generated under the normative condition as normative labels.

### Descriptive and normative labels are significantly different

We first tested the hypothesis that descriptive and normative labels are drawn from the same distribution. We began by constructing a single judgment label for each object by averaging the judgment labels from the 20 labelers for each condition. This yielded one normative and one descriptive label per object: the percentage of participants whose answers generated a judgment label of “violation” under that condition. The label 1 represents complete labeler consensus that the object violates the relevant code, and the label 0 represents complete consensus that there is no violation.

**Fig. 3.**
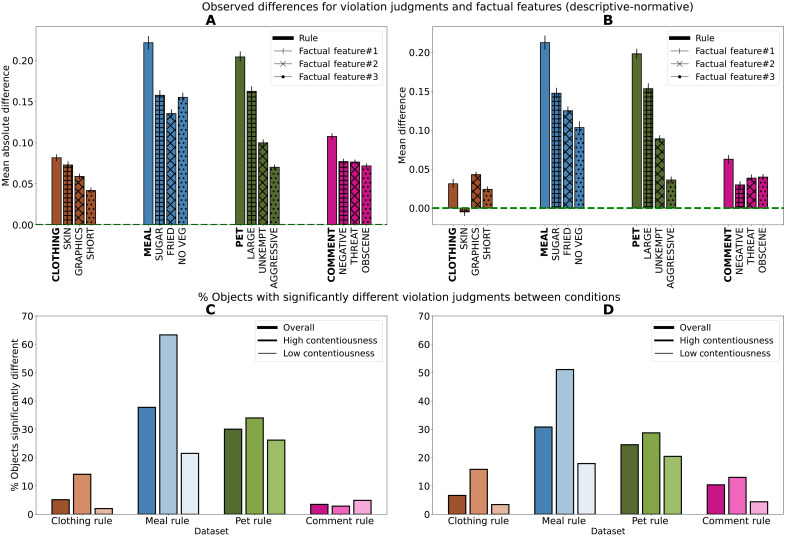
Judgments generated with descriptive labels are significantly different from those generated by collecting normative judgments. We examine the difference between labels collected under the descriptive and normative conditions with (**A**) the mean absolute difference between labels that say violation in each condition and (**B**) the mean difference between the same, the direction of which shows that violation labels in the descriptive condition are often higher. We measure the proportion of objects where the distribution of labels collected is significantly different between (**C**) the two conditions. This proportion is also assessed between (**D**) normative and descriptive-with-context labels.

We find strong evidence (Fig. 3A) to reject the hypothesis that the judgment labels generated under the two conditions are drawn from the same distribution in all four settings (*P* <0.0001, Kruskal-Wallis *H* test; exact test results in Supplementary Text). This difference is large: The mean absolute difference (i.e., the absolute average difference in the percentage of labelers who identify a violation between the two conditions) ranges from ≈10% in Clothing and Comment datasets to 20% in the Meal and Pet datasets.

The difference between conditions is also mostly consistent in direction (Fig. 3B). For each setting, the average descriptive label for an object is significantly higher than its corresponding average normative label. This means that an object is significantly more likely to be labeled as a violation in the descriptive condition than in the normative condition (*P* <0.0001, one-sided Wilcoxon signed-rank test). The magnitude of this difference in the Clothing and Comment datasets is less than 8%, increasing to between 15 and 20% in the Meal and Pet datasets. Note that here we discuss the mean difference, whereas in the previous paragraph we discuss the mean absolute difference. Unpacking these overall judgments, we also compared labels given for the underlying factual features in each condition, again averaging across the 20 labelers in that condition. We find that for 11 of the 12 features within the four rules, the labels generated in the descriptive condition are higher than those in the normative condition (Fig. 3B). This confirms that even when asked to focus on the presence of features, participants were less likely to find that a feature is present in the normative than in the descriptive condition.

Given the variation we observe between normative and descriptive labels, we examined whether some objects would be labeled significantly differently under the two conditions. To estimate this, we conducted a statistical test between the binary violation judgments collected under the two conditions for each object (using Boschloo’s exact test, a variant of Fisher’s exact test). When this test returns a *P* value of ≤0.05, the conditions are significantly different on that object. In all four settings, we find that the percentage of objects that receive significantly different judgment labels between the two conditions ranges from a low of 3.5% (Comment) to a high of 37.8% (Meal) (Fig. 3C).

### Labeling differences are robust to example contentiousness

In the previous section, our analysis focuses on statistics that are averaged across judgments from all objects in our dataset. But many of these objects have low contentiousness: They clearly do or do not represent violations of the relevant rule. We expect high labeler agreement in these cases, and we might expect that there would be no difference between the descriptive and normative conditions. For example, an image of a person wearing full-length trousers, a buttoned-up shirt, and a suit jacket is not likely to be labeled as a violation of our dress code, in either condition. The cases in which we might see a difference in normative judgments are likely to be highly contentious, in which judging the presence of a feature even as a factual matter is reasonably subjective: How short does a skirt have to be before it is judged to be “short”? These are the cases for which normative judgments about violations could be challenged and require justification. In practice, it is these highly contentious cases where normative judgments are more likely to be challenged, requiring justification. In these cases, we expect higher rates of labeler disagreement, and we would predict more opportunity for differences in normative judgments to emerge across our conditions. We heuristically define an object to have high contentiousness if it has at least 20% disagreement among its descriptive labels.

Using the same methodology as the previous statistical test (Boschloo’s exact test), we find that the percentage of objects with high contentiousness that receive significantly different judgment labels between the two conditions ranges up to 63.32% (Meal) (Fig. 3B). That is, in most cases, objects with high contentiousness are more likely to receive labels that are significantly different between the conditions than other objects. Judgment labels are significantly different between the two conditions for 4.91% of the text objects with low contentiousness.

**Fig. 4.**
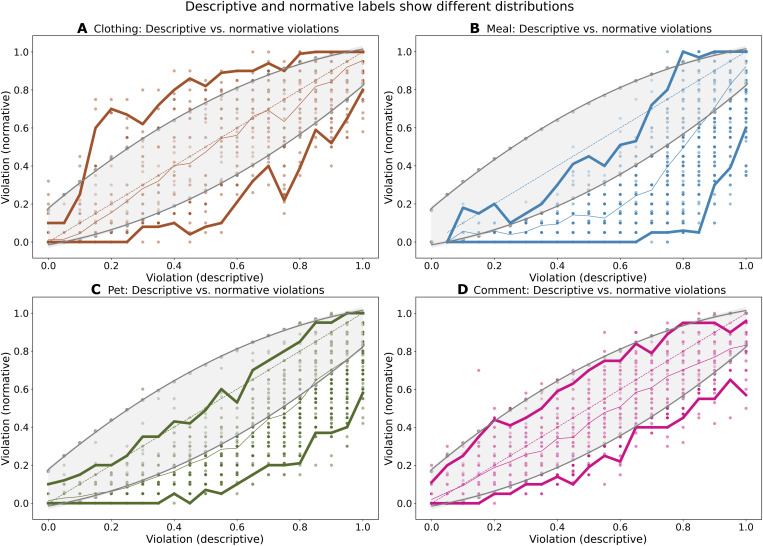
Observed normative judgments deviate from what we would expect if the normative and descriptive conditions were identical. The plot above shows the distribution of normative labels for each descriptive label value (rounded) for the (**A**) Clothing, (**B**) Meal, (**C**) Pet, and (**D**) Comment datasets. The scattered points show the normative labels obtained for each unique descriptive violation label estimate. We observe that normative judgments deviate frequently from the 45° line that we would expect to see if the two conditions were identical. Gray lines and gray shaded regions indicate 95% Clopper-Pearson intervals around the descriptive label point estimate, and the colored curves in each case connect the medians and 95% confidence intervals of normative labels. We see that several points lie outside the gray shaded region, interpreted as normative labels are frequently outside what we would expect given the descriptive labels, and their associated variance. Usually, this deviation is downward: Observed median for normative judgments is frequently lower than the corresponding descriptive judgments for Meal, Pet, and Comment datasets.

Furthermore, we note that descriptive-normative label differences in individual object labels are present across a range of labeler disagreements (Fig. 4). An automated decision system outputs a binary label—violation or not—based on a defined threshold proportion of labelers who label an object as a violation. For example, at a low threshold, even a small number of labelers judging an object to be a violation will be sufficient for the system to label it as a violation. We find that the difference we identify persists across a range of plausible thresholds for deriving such a binary label (fig. S2).

### Labeling differences are robust to judgment context

One possible explanation for the difference observed between normative and descriptive conditions could be that normative condition participants simply have more context. For instance, “high skin exposure” could be evaluated differently if participants think of this feature in the context of an outfit suitable for going to an office rather than for going out with friends. This could mean that the difference we observe in labeling does not stem from how people judge facts versus norms, but rather from the information they have available when making a subjective factual judgment.

To investigate this possible effect, we introduced a third condition: descriptive-with-context, which is identical to the descriptive condition, except with additional context about the situation. For Clothing, we specify that we are asking participants to identify features to evaluate whether clothes “could be worn in an office or school setting”; for Meal, whether meals are “healthy and wholesome”; for Pet, whether dogs “would not be out of place in small indoor spaces”; and for Comment, whether a text snippet is “respectful.” We find that even with this added context, the descriptive and normative settings are significantly different on average (*P* <0.0001, Kruskal-Wallis *H* test). In addition, for two of the four settings (Meal and Pet), the descriptive and descriptive-with-context settings are not significantly different on average—Meal: *P* = 0.22 and Pet: *P* = 0.08 (Kruskal-Wallis *H* test).

The descriptive-with-context and normative conditions also produce significantly different judgment labels for as many as half of the objects with high contentiousness (Fig. 3). Notably, introducing context in the Comment dataset increases the number of examples showing a significant difference between descriptive and normative labels from about 4 to 10%. We conclude that the effects we observe are not driven by variation in the availability of context for the judgment labelers are asked to make.

### Labeling differences impact downstream model performance

Our labeling experiments and analyses demonstrate significant differences in descriptive and normative judgments. Here, we study the impact of these labeling differences on the performance of ML models trained to automate normative judgments. This aims to determine how accurately models trained to judge facts (i.e., trained using descriptive labels) predict judgments about rule violations (i.e., normative labels). In the language of ML, we are testing the performance of descriptively trained models under distribution shift (*24*), specifically, a shift in label distribution from descriptive to normative labels.

For each dataset, we train models using either the descriptive or normative labels and test them on a set of held-out data with normative labels. In all cases, we train supervised multitask prediction models where the (constructed) descriptive or normative rule violation judgment, as well as the three factual feature values are predicted (fig. S3). ResNet-50 (*12*) and ALBERT (*13*) models are used for image and text classification, respectively. Experimental details are in Supplementary Text.

**Fig. 5.**
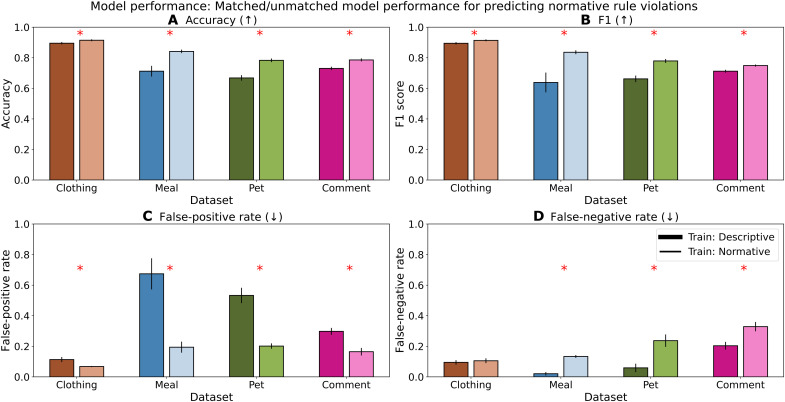
Models trained on descriptive labels result in statistically significantly different predictions from models trained on normative labels. Training models on descriptive labels (Train: Descriptive) yields significantly lower accuracy and higher false-positive rates (i.e., incorrectly labeled as a violation) on the four settings, when tested on held-out objects with normative labels (Kruskal-Wallis *H* tests; also see fig. S4). Whiskers indicate 95% confidence intervals for each performance metric (**A** to **D**). Arrows in each plot title indicate direction of “better” performance.

We find that models trained using descriptive labels achieve lower accuracy (Fig. 5) in predicting normative rule violations, especially on objects that are difficult for humans to agree on normatively. Even with relatively small datasets (2000 objects)—where sampling variance can yield noisy results—this difference in accuracy/F1 score is still significant (*P* ≤ 0.05, one-sided paired Wilcoxon signed-rank/Kruskal-Wallis *H* tests). We note that models trained on the Comment dataset have relatively high variance in performance [as observed in (*25*)], which is why despite a persistent gap, the difference in accuracy may not be statistically significant with a paired test for some models (while significant with an unpaired Kruskal-Wallis *H* test; see Supplementary Text). Furthermore, we find that descriptive models are associated with a higher false-positive rate on the test set in each case; that is, they are more likely to wrongly call an object a violation. Hence, more violations in the data predictably yield more predictions of violation outputted by the model (see Fig. 3, where descriptive labels are more likely to generate a judgment label of violation). Higher differences and similar trends were observed on objects that are difficult to agree on normatively; that is, those that exhibit high contentiousness in their average normative judgment label (e.g., fig. S4).

**Fig. 6.**
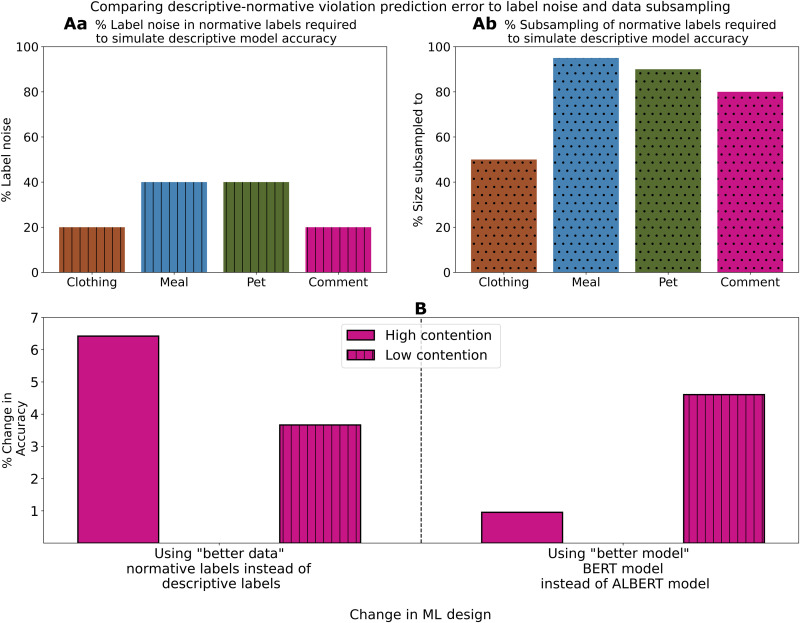
The impact of the descriptive-normative measurement error is comparable to large changes in data noising, data size reduction, and model class change. Increase in classification error incurred by switching from normative to descriptive labels at training time is comparable to (**Aa**) adding 20% to 40% label noise or (**Ab**) reducing the dataset size by 50 to 95% (top left and top right). (**B**) Furthermore, gathering better data (using normative labels on normative task with the same model capacity) affects classification accuracy comparably to training a better model (using descriptive labels on normative task with a higher model capacity; bottom).

We contextualize the size of the loss in accuracy by comparing it to equivalent changes in accuracy caused by label noise, less data, and changes to the model architecture. In Fig. 6A, we show that the average accuracy loss caused by a switch from normative to descriptive labels is equivalent to adding 20 to 40% label noise at training time or to removing 50 to 95% of the training and validation data points (with the same hyperparameter configuration). In Fig. 6B, we compare with a change in model architecture from a small distilled transformer model “ALBERT” (*13*) to a large transformer model “BERT” (*26*). When both models are trained with descriptive data, the larger model’s accuracy improves by 2.12% on the Comment dataset (4.61% increase on objects with low contentiousness; 0.96% increase on objects with high contentiousness). In contrast, training the smaller ALBERT model on normative labels improves performance by 5.54% (6.42% on objects with high contentiousness; not significantly different with Kruskal-Wallis *H* test at 0.05 level with correction for multiple testing). These model architecture choices are often highly optimized (*27*, *28*) by ML researchers and practitioners. For example, model class difference between RoBERTa (*29*) and ALBERT leads to 0.5% accuracy difference on the MNLI-m (*30*) benchmark. This suggests that better labeling choices have a relatively high impact on the performance of ML models in normative applications. We observed, additionally, that relative model performance ordering of BERT and ALBERT models varies across random initializations.

Furthermore, preliminary analysis in the Comment setting indicates that the impact of rule-prediction errors due to descriptive-normative label differences might vary across subgroups. For example, the difference in predicted rate of online forum rule violations with descriptive and normative models for comments with male mentions is 13.48% with descriptive model and normative models, in comparison to 14.55% for comments with female mentions.

Last, as a robustness and generalizability check, we verified that observed trends of higher violation rates with descriptively trained models persist on a closely related large preexisting benchmark datasets in the Comment setting (section SA.11).

## DISCUSSION

We show that directly elicited rule violation judgments significantly differ from those constructed by identifying the presence of the factual features of a rule. This is a surprising result. Logically, we would expect these two procedures to produce the same result.

We note that the factual features we presented often require subjective decisions: Is skin exposure in this outfit high? Does this comment contain obscene language? These are not factual assessments with objectively correct answers. The degree of subjectivity may vary across factual features. We see variance in the assessment of the facts in our descriptive condition. But this variance in the assessment of facts does not explain the gap between judging facts and judging norms. If only subjectivity were at work, we should see the same proportion of participants judge a feature to be present in a given object as judge a rule to be violated because the feature is present. Our results, however, show a difference (Fig. 3A) in these proportions: consistently smaller proportion who identify an otherwise ambiguous feature to be present when asked to judge in the normative condition (as opposed to descriptive condition) (Fig. 3B).

Our modeling results indicate that the difference between descriptive and normative labels propagates through to model performance. Specifically, models trained using descriptive labels (i.e., constructed violation judgments) underperform in comparison to those trained on normative labels. We propose that this can be seen as an important form of measurement error in the context of automated decision systems. In such systems, we seek to automate the judgments that humans reach. The correct output label would therefore arguably be the label generated by participants in our normative condition: Does this object violate the rule? We show that a model trained on factual feature labels generated in a descriptive condition will consistently get this wrong, that is, an automated system will judge differently from humans.

In related work, Rottger *et al*. (*11*) also draw attention to the importance of paying attention to how humans, in practice, label objects (in their case, text) for the purposes of automating judgments. Like us, they propose a distinction—between a descriptive paradigm and a prescriptive one—and conduct a human subjects experiment to identify differences in labels arising from different labeling instructions. They also emphasize the importance of aligning label collection practices with the purposes to which a model will be put. Their focus, however, is on the extent to which a model aims to elicit (descriptive) variation in individual beliefs (about what constitutes hateful speech, for example) versus focusing labelers (prescriptively) on a single, prescriptive belief (about what constitutes hateful speech according to a social media platform’s detailed content guidelines, for example). In our framework, both of their paradigms are “descriptive” as they elicit assessments about (subjective) facts; their “prescriptive” paradigm does not include our “normative” condition in which labelers are asked to exercise direct judgment about violations of (for example) a content policy.

### Labeling differences affect automated judgments

The data selection and labeling processes for automated systems are often not fully public and may involve repurposing of historical data (*31*). From the limited disclosures available, we were able to identify a few examples of descriptive labeling practices for normative applications (*19*, *20*). While there are no publicly available examples demonstrating the automation of normative judgments based on factual labels alone, some examples, nonetheless, reveal an implicit assumption that building a system to predict factual evidence is an appropriate approach to supporting human judgment (as opposed to building a system to predict human judgments of factual evidence). Law enforcement, for example, is making use of ML models that make factual predictions about the content of videos to assess violations of child pornography laws (*32*), and courts are using models that make factual predictions about the likelihood a defendant will reoffend to reach normative judgments about bail, sentencing, and probation (*31*, *33*, *34*). We also note that even when researchers do pay close attention to labeling practices, and subjectivity in particular, there continues to be a seemingly natural assumption that judging the presence of factual predicates is appropriate for training a model to exercise judgment (*11*).

Our result exposes important considerations for the application of ML to normative settings. In settings where the goal is to scale existing normative processes, machine judgments may introduce a new source of bias. We found that automating decisions with a model trained on descriptively generated labels resulted in judgments that were harsher in comparison to directly elicited human normative judgments (for the particular settings and participant group we studied). This can have real consequences for people who are judged by machines, and they may be likely to object to having their case decided by a machine.

In other cases, the goal of an automated system is to mitigate human failures of judgment, such as racial or gender bias (*35*). In these cases, it may be tempting to think that a “just the facts” approach to constructing machine judgments would be preferable to training on biased human judgments. Our results demonstrate a flaw in this approach. Bias does not appear to explain the difference between our descriptive and normative conditions: Two of our settings—Meal and Pet—do not raise a risk that judgments will be significantly distorted by biases against protected groups. Furthermore, factual predictions can introduce bias: The widely debated COMPAS system for predicting recidivism risk, for example, uses a biased proxy for reoffending-rearrest—to train its models (*36*). Our work suggests that attempts to reduce bias should focus on improvements to the process that generates labels. For example, instead of replicating historical decisions, one might attempt to curate data for normative judgments, based on judgments elicited in idealized settings that manage or mitigate bias. Models trained on these data avoid the trap of false objectivity. This is much more expensive than training on the actual data generated by human judgment systems, but we believe that this is what is required by fairness and fidelity to the difference between how humans judge facts and how they judge norms.

### Broader connections in legal and psychology literature

Our results resonate with effects found in related experiments in the psychology literature. Tversky and Kahneman (*37*) first drew attention to the phenomenon of framing, showing that individuals vary in their tolerance for risk depending on whether a gamble is presented as a loss or gain. Numerous studies have since shown that choice varies depending on whether it is presented in a positive or negative frame (*38*, *39*). We verified that this specific framing effect was not responsible for our results. In a robustness check reported in Supplementary Text, our normative tasks framed as “compliance” instead of violation did not lead to statistically significant differences (Kruskal-Wallis *H* test on a representative subsample of our datasets).

Other studies in law and psychology have investigated aspects of the relationship between factual and normative judgments, and in particular the impact of sequencing. In a classic study, Simon and Mahan (*40*) found that jurors were less likely to find a defendant guilty in a criminal case if they were first asked (before deliberation with others) to give their estimate of the likelihood that the defendant, as a factual matter, committed the criminal act. In another important set of studies, Holyoak and colleagues (*17*, *41*, *42*) show that reported factual assessments can be influenced by the subsequent exercise of normative judgment, with initially mixed perceptions of facts shifting to more extreme assessments that support emerging judgment. This effect is seen as reflecting a weakness in human normative reasoning, as people alter their beliefs about factual matters to achieve greater coherence between their beliefs and their judgment. Like our study, this research shows that judgments about facts (and legal conclusions) can vary depending on whether someone understands themselves as being asked to evaluate a set of circumstances as a descriptive matter or to exercise judgment.

Our results are not, however, explained by the effect found in these papers. In another robustness check, we found no significant difference between judgments of violation elicited before and those elicited after assessments of the presence of facts supporting the finding of violation (Kruskal-Wallis *H* test with multiple-testing correction on a representative subsample of our datasets, reported in Supplementary Text). In addition, from a theoretical perspective, there is little reason to think that our results are driven by cognitive dissonance and the drive to coherence that the law and psychology literature has identified. The coherence studies in (*17*, *41*, *42*) examine complex judgment settings—such as criminal trials—in which substantial amounts of ambiguous evidence are heard over time and then judgment is reached. The coherence effect suggests that humans in this setting may begin to form normative conclusions (guilty, not guilty) before they have heard all the evidence and then may alter their perceptions of the evidence to support their emerging judgment. The study in (*40*) considers judgments made after deliberation with others and the impact of assessing facts before deliberation. In our study, we have a simple rule, no deliberation, and no substantial time lapse between making a judgment and offering factual reasons (judgments and reasons are elicited in a single survey screen). In addition, our participants are not changing their factual judgments following normative judgment—the differences we see are between subjects, not within subjects.

Our work is also closely tied to language model–based computational ethics research. However, while a majority of recent work has focused on building models that have the capacity to make human-like moral judgments (*43*, *44*), our study investigates a core assumption about human normative reasoning inherent to current ML data acquisition practices.

### Data labeling for normative tasks requires careful attention in supervised machine learning pipelines

We emphasize that we are studying alternative approaches to labeling for ML that produce desired outcomes, not human psychology per se. We are comparing an approach that appears sensible—collect factual labels and then use them to construct judgments—with an alternative that we might predict is equivalent—ask labelers directly to exercise judgment—and demonstrating that these are not equivalent. The law and psychology literature identifies errors that human (individual and collective) reasoning in complex settings might make and offers proposals to mitigate these errors. Our participants are not obviously making errors of judgment. They may simply be engaging in different cognitive processes when they make normative judgments than when they evaluate facts. This is a hypothesis not explored in the existing law and psychology literature. But the hypothesis that judging facts and judging norms are just different tasks altogether is plausible when we think about what humans do when judging norm violations. Experimentally, we consistently find that our participants give the object being judged the benefit of the doubt: A close call is often judged to be a nonviolation. One interpretation is that our participants attach a different cost to their judgments in the two conditions: Getting a decision wrong factually is just a matter of describing the world incorrectly. Getting it wrong normatively is a matter of potentially doing harm to another human. In statistical terms, our participants may evaluate false positives and false negatives equally in the descriptive setting, but place greater weight on false positives in the normative setting.

We suggest that it is an open question whether the “correct” way to exercise normative judgment is to evaluate facts first and then apply logic to those facts, or to state a judgment and then articulate the factual justification for that judgment, particularly when facts are ambiguous. Many systems of human judgment, in practice, do not force a commitment first to factual findings and then an exercise of normative judgment. Usually, juries are not asked to first report back on their factual findings and then their judgment of “guilty” or “not guilty.” Although the U.S. Supreme Court does not decide factual matters, the procedure for deciding cases is for the justices to first vote on the outcome and then for an assigned justice to produce the reasons to justify that outcome. Many administrative decisions are made (tax deductions allowed or denied, health and safety regulations deemed violated or not, for example) without reasons at all in the first instance; reasons are only produced ex post if the decision is challenged. The fact that so many human judgment systems take this approach is not obviously a mistake we should correct with automation.

Our findings explicitly sharpen the question of “who” should exercise judgment. If descriptive labels do not translate logically into normative judgments, we should expect such human judgments to vary across groups, cultures, geography, and time. Having representative group participation should be a topic of consideration in the public oversight of ML systems. A limitation of our setup is that all participants in our study were recruited via Amazon MT. Because of the nature of tasks frequently seen on the platform, these participants may exhibit relatively lower variability in specific labeling contexts in comparison to general rater pools. Validating the generalizability of findings in different expert and non-expert populations might thus be an important direction for future work. Controlled experiments assessing the impact of the degree of subjectivity inherent to factual features in each case are also an important direction for future work.

### Implications for responsible AI development

There are important practical takeaways from our results for responsible artificial intelligence (AI) development. These takeaways augment research on fairness in AI systems and social biases inherent in data labeling (*45*, *46*).

We propose that dataset and model producers who create or procure datasets to train ML systems should disclose the labeling prompts used for data collection (e.g., descriptive versus normative framework) and explicitly state to which potential normative use cases their models best apply. For instance, a model trained on a dataset of images gathered with descriptions of content or quality could include a statement that the model is not necessarily well suited to normative applications that rely on the learned descriptions. This could serve as a warning that there are likely to be performance gaps between descriptive and normative test cases. As a goal, dataset and model producers should aim to train on normative labels elicited under carefully curated ideal conditions for normative tasks. At a minimum, there should be disclosure if a model was trained on descriptive labels, disclosure of the nature of the test set, and a warning that a model trained on descriptive labels may underperform on normative tasks. Dataset and model producers should use tools like Datasheets for Datasets (*47*) and Modelcards for Model Reporting (*48*) for increased transparency.

As for model users, we urge them to ensure that models deployed to perform normative tasks are either trained on carefully elicited normative labels or appropriately adjusted to address performance gaps. If the training data for a model are not clearly disclosed, users should request specifics of the prompts and contexts used to generate the model’s training data labels. Crucially, our work suggests that downstream users may need to know details about the framing used for data collection. The simplest intervention users can adopt is to use a normative test set that is specific to the deployment context. A potential remedy may be to fine-tune models on normative labels as an internal modification to be applied before any broad use. In addition, the interplay between the perceived legitimacy in end-users affected by automated judgments and the data labeling prompts used must be studied.

Last, from a regulatory and auditing perspective, it is clear that far more attention needs to be paid to how the data on which automated decision systems are trained are selected and labeled. Although we join the calls for ensuring that datasets are unbiased, our work shows that debiasing data will be insufficient to ensure that the results of automated systems are a faithful implementation of the rules and norms that govern our societies. As we noted above, obtaining the judgment labels that reflect ideal human judgment may well require expensive curation of task-specific data, informed by a social scientist’s attention to the details of how judgments are elicited. Regulation may well be necessary to overcome the powerful incentive at work in today’s ML ecosystems to rely on cheaply available prelabeled datasets.

One of the aspirations of automated decision systems can be to improve on flawed human judgment. Our results highlight the complexity and potential pitfalls of this ambition. They suggest that increased attention and care should be paid to the normative process being augmented or automated. Developers should be explicit about the goals of automation, ground their methods in existing human judgments, and disclose the details of the normative process that is being implemented. Our results undermine the notion of the so-called “superhuman” performance in normative applications. Instead, attempts to mitigate flawed judgment should focus on curating data from judgment processes that are designed to directly mitigate unwanted bias.

Last, we emphasize that while we purposefully chose decision-making settings that are realistic in our study, we do not advocate that these be used in practice. Using AI tools for detecting rule violations is a complex topic, and automated decision-making may be inappropriate in some settings. We have demonstrated that such violation detection may not reflect human judgments, and prior research has shown that deployed AI systems are prone to failures and biases (*49*). Furthermore, human operators may follow biased advice (*50*) from such models. Many hard human problems may not have technical solutions. We stress that deployment of models in human systems must carefully consider the full societal context.

## MATERIALS AND METHODS

### Participants and recruitment

We recruited participants using Amazon MT (https://requester.mturk.com) with the following inclusion criteria: (i) participants reside in either the United States or Canada and (ii) each participant has an approval rating over 98%. The project was approved by the University of Toronto’s Institutional Research Ethics Board (protocol no. 00037283).

We created tasks where participants provided labels for the four different datasets, each containing 2000 objects and three treatment conditions. Recruitment for each of the four datasets was performed independently with an online controlled experimentation approach. Given the large number of objects to be labeled (2000 data points to label per condition in each dataset), we created streaming labeling jobs wherein we sent new dataset objects to participants in real time by running labeling jobs over time. Every such job is a microtask or a Human Intelligence Task (HIT; https://www.mturk.com/help), consisting of instructions and objects to be labeled. Each dataset object—embedded in HITs—was labeled by 20 different participants in each condition and dataset.

Participants continually received new data objects to label as long as the labeling HIT was active, and they had the necessary qualifications to complete the job. Note that the order of such HITs visible to participants is randomized by default (implemented by Amazon MT). As a result, the size of the participant pool increased over time for each condition within each dataset. Additional screening was performed using input provided to attention checks embedded within each HIT. If a given job was rejected (e.g., due to failing an attention check), the job reentered the stream of objects to be labeled and was picked up to be labeled by another participant. Jobs were released in batches, in accordance with best practice guidelines for data labeling on MT, to impose an upper limit on the number of HITs an eligible participant can complete at a given time (i.e., the number of unique HITs in a batch). We observed that the total number of HITs completed by a participant (i.e., across batches) varied depending on the task.

The four major dataset collection studies were completed between May 2020 and July 2022. Some portion of Pet and Comment dataset collection overlapped, while other datasets were collected sequentially to minimize cross-dataset overlaps. In total, we recruited 3373 participants across all four datasets. Details of the participant population are in Table 1.

**Table 1.** Population details.

Dataset	Condition	*N*	Gender		
			Female	Male	Other
Clothing	Descriptive	439	79	61	0
	Normative	601	105	154	1
	Descriptive-with-context	269	43	56	2
Meal	Descriptive	241	78	113	1
	Normative	262	115	98	3
	Descriptive-with-context	206	57	114	1
Pet	Descriptive	402	91	94	4
	Normative	573	118	105	3
	Descriptive-with-context	204	111	91	2
Comment	Descriptive	268	93	119	1
	Normative	258	90	114	3
	Descriptive-with-context	206	61	78	0

We estimated the completion time taken and clarity of instructions for each HIT via pilot studies (details in section S5.5). We compensated each HIT with the aim to pay at least $12 per hour. In general, we observed that completing the normative labeling task took longer and, hence, paid slightly more to reflect this. In total, for the four datasets, we paid $5140.24 for the Clothing, $5944.24 for the Meal, $7123.98 for the Pet, and $5616.1 for the Comment dataset labels, all not including tax.

### Annotator label collection details

In fig. S1, we show an example interface from our MT data collection pipeline. This contains three key elements:

1) An introductory prompt: In the descriptive condition, we said: “We would like you to identify some attributes of...,”, and in the normative condition, we introduced and described the relevant code or policy and asked the annotator to make a judgment of violation/no violation.

2) The object(s): We displayed the object in question one after the other, whether an image or text snippet.

3) The answers below each object: In the normative condition, we first asked the annotator to make a yes/no judgment about whether the code is violated, and then asked for each of the three reasons (i.e., factual features) to justify their decision with yes/no choices. In the descriptive condition, we only asked for yes/no answers for the three factual features as to whether the attributes are present.

The descriptive-with-context condition is identical to the descriptive condition, with slightly modified introductory prompts. Screenshots of prompts for all conditions for all datasets can be found in the Supplementary Materials.

### Data source and sample curation

We curated examples to create four datasets from the following sources:

1) Clothing—2000 images sampled from ClothingAttributes (*51*) and DeepFashion dataset (*52*) (with all faces in images blurred out to protect privacy).

2) Meal—2000 images sampled mostly from Food-101 (*53*), Food-11 (*54*), and FFoCat datasets (*55*). Some images of breakfast cereals were additionally chosen from Pixaby (https://pixabay.com/) for the “high sugar content” category by using the search terms like “breakfast” (due to few images of this type in the two datasets). We also selected few images from open-sourced Kaggle (https://kaggle.com/datasets) datasets.

3) Pet—2000 images sampled from the Stanford Dogs (*14*, *56*) and Dogs vs Cats Kaggle Asirra dataset (*57*).

4) Comment—2000 comments sampled from the CivilComments (*58*) training set.

We selected 2000 unique samples by inspecting a large random sample from each of the datasets listed above and curating objects in which at least one factual feature (see Fig. 2A) in each setting could arguably be present. Data sample selection and curation were performed in batches, in parallel to data labeling studies on MT for all four datasets. Data samples were selected with the aim of measuring differences between descriptive and normative labeling procedures, all with the criteria that the three factual features can reasonably be inferred. As a result, our datasets are not truly random or representative subsets of the large datasets they are selected from (see Supplementary Text for per-dataset sample selection details).

### Dataset-specific code construction

We created hypothetical codes or policies governing four settings. Each code follows a similar structure, containing three factual features. An object is in violation of the code when it contains any of these factual features. These codes are described here:

1) Dress Code (“Clothing”): We used images of people wearing various outfits and created a dress code in which someone is in violation of the dress code if they are deemed to be wearing clothes that (i) show high skin exposure; (ii) contain text, graphics, or any images; and (iii) contain short shorts or short skirt.

2) School Meal Restrictions (“Meal”): We used images of various foods or meals and created a school meal policy in which a meal is considered unfit to be served in the school if it is deemed to (i) have high sugar content, (ii) contain a significant amount of fried food, and (iii) contain less than a full serving of fruits and vegetables.

3) Apartment Pet Code (“Pet”): We used images of dogs and created a pet code for an apartment building in which a dog is considered unsuitable for apartment living if it is deemed to (i) be large sized, (ii) be not well groomed, and (iii) appear aggressive.

4) Discussion Forum Comment Guidelines (“Comment”): We used comments from Internet comment boards and created a discussion forum guidelines in which a comment is not allowed to remain on the forum if it is deemed to (i) contain negative comments about race, sexual orientation, gender, religion, or other sensitive personal characteristics; (ii) be threatening to a person, group, or organization; and (iii) use obscene language. In all cases, the factual features were identified by surveying real examples of dress codes, school meal policies, building pet policies, and online discussion forum guidelines.

### Study design

Our study design is between-subject with assignment to one of the three treatment conditions for each dataset. Hence, once participants completed a labeling task for a given condition and dataset (e.g., normative dress code labeling), they are assigned qualifications via MT to block them from being able to complete HITs for other conditions for the same dataset. Specifically, this was performed by consecutively sampling participants from the MT population by releasing batches of jobs for each condition one after the other and assigning qualifications. With this manner of recruitment, common to most ML labeling tasks on crowdsourcing platforms, the number of participants recruited for each condition for each task is in Table 1.

For example, for the Meal dataset, we released most HITs in batches of 200 images each for the three conditions within 1 to 2 days of each other. The timing does not matter, because the variables of interest (factual features and judgment) do not depend on time of labeling. We note that data collection for the context setting in the Clothing dataset was not performed in this manner.

The requester is blinded to MT worker details (anonymized IDs and no demographics-based selection); hence, there is no risk of selection bias. We also collected demographic information related to the age (18 to 29, 30 to 49, 50+) and self-reported gender (Male, Female, Other-specify) of participants (descriptive statistics of the self-reported gender in Table 1). However, this is available only for a subset of participants, as demographic collection was gathered as a follow-up survey to annotators or a pre-survey before HITs (also via MT). We show in Supplementary Text that the descriptive versus normative label difference is not driven by demographic differences and that the group assignment—descriptive versus normative—significantly affects the labels even when controlling for demographic variations.

We ensured that any cross-dataset condition overlaps that occurred does not cause the main results to vary via robustness checks in Supplementary Text. We also used good practices for high data quality (for example, see https://cloudresearch.com/resources/blog/best-practices-online-research-mturk/) (see Supplementary Text) by reviewing the data using the Amazon MT review interface.

### ML model training

We trained multitask prediction models where both the code/policy violations and the individual attribute (factual feature) values are predicted jointly. Hence, the training data consisted of tuples of the form (*x_i_,y_i_*), where *y_i__i_* = [*y*_*i*0_, *y*_*i*1_, *y*_*i*2_, *y*_*i*3_], *y*_*i*0_ refers to the code/policy violation label, and *y_ij_* refers to the *j*th factual feature for the *i*th sample point. We did not aggregate labels for each image/text object in the training and validation set to increase the size of training data points and to avoid having to preselect thresholds before training models.

Hence, the size of the dataset for each task is 40,000 (2000 objects with 20 labels per object). A given dataset is randomly split into ≈60% train, 10% validation, and 30% test set splits, where we ensured that the proportion of objects with high contentiousness (that are likely more difficult to classify correctly) in each split is approximately the same as that of the full dataset. All models are trained with a cross-entropy loss for jointly predicting the code/policy violation label and corresponding factual features. Thus, the loss function optimized is the sum of cross-entropy loss for the four prediction outputs with respect to train targets, each equally weighted (i.e., a loss weight of 0.25 or 1 for each term). In the subsections below, we describe ML model implementation details for image and text settings.

#### Image

For all of our reported results, we used the ResNet-50 (*12*) model pretrained on ImageNet (*14*). We added a multitask head to the pretrained model to jointly predict the factual features and the code/policy violation. We tuned hyperparameters of batch size, learning rate, and weight decay using grid search based on violation classification performance on the validation set (search space of *{32,64,128}, {0.1,0.01,0.001}* and stepwise linear learning rate decay with gamma in *{0.1,0.5}*, respectively). All models were fine-tuned up to 20 epochs. To obtain predictions for the test set, we used the model weights from the epoch with the highest validation F1 score (macro-averaged).

#### Text

For all our main reported results, we used the ALBERT model (*13*) pretrained on CivilComments. We added a multitask head to the pretrained model to jointly predict the factual features and the code/policy violation. We also use a pretrained BERT model (*26*) in some experiments to compare the effect of different design choices on the violation prediction performance. Note that on the benchmark Jigsaw CivilComments challenge task, larger BERT-based models slightly outperform ALBERT-based models (*58*), but on MNLI (*30*), the opposite trend in model performance is observed. Following prior work (*59*), we set the batch size and learning rate to 32 and 3 × 10^−5^, respectively, for tuning the language model–based text classifier. All models are fine-tuned up to 10 epochs, and the epoch with best validation F1 score (macro-averaged) is selected for testing. Additional details of the final hyperparameter settings and performance metric computation for all models are described in Supplementary Text.

## References

[1] K. A. Houser, Can AI solve the diversity problem in the tech industry: Mitigating noise and bias in employment decision-making. STLR 22, 290 (2019).

[2] M. Bergius, E. Ernberg, C. Dahlman, F. Sarwar, Are judges influenced by legally irrelevant circumstances? Law Probab. Risk 19, 157–164 (2020).

[3] J. Kleinberg, H. Lakkaraju, J. Leskovec, J. Ludwig, S. Mullainathan, Human decisions and machine predictions. Q. J. Econ. 133, 237–293 (2017). 29755141 10.1093/qje/qjx032 PMC5947971

[4] A. S. Wicaksana, C. C. Liem, Human-explainable features for job candidate screening prediction, in *2017 IEEE CVPRW* (IEEE, 2017), pp. 1664–1669.

[5] J. Galindo, P. Tamayo, Credit risk assessment using statistical and machine learning: Basic methodology and risk modeling applications. Comput. Econ. 15, 107–143 (2000).

[6] D. F. Engstrom, D. E. Ho, C. M. Sharkey, M.-F. Cuéllar, *Government by Algorithm: Artificial Intelligence in Federal Administrative Agencies* (NYU School of Law, Public Law Research Paper, 2020).

[7] M. Kuziemski, G. Misuraca, AI governance in the public sector: Three tales from the frontiers of automated decision-making in democratic settings. Telecomm. Policy 44, 101976 (2020). 32313360 10.1016/j.telpol.2020.101976 PMC7164913

[8] G. Malgieri, Automated decision-making in the EU Member States: The right to explanation and other “suitable safeguards” in the national legislations. CLSR 35 105327 (2019).

[9] L. Morawski, Law, fact and legal language. Law Philos. 18, 461–473 (1999).

[10] M. C. Redondo, *Reasons for Action and the Law* (Springer Science & Business Media, 1999), vol. 43.

[11] P. Rottger, B. Vidgen, D. Hovy, J. Pierrehumbert, in *Proceedings of the 2022 Conference of the North American Chapter of the Association for Computational Linguistics: Human Language Technologies* (Association for Computational Linguistics, 2022), pp. 175–190.

[12] K. He, X. Zhang, S. Ren, J. Sun, Deep residual learning for image recognition, in *IEEE Conference on Computer Vision and Pattern Recognition* (CVPR, 2016), pp. 770–778.

[13] Z. Lan, M. Chen, S. Goodman, K. Gimpel, P. Sharma, R. Soricut, ALBERT: A lite BERT for self-supervised learning oflanguage representations, in *International Conference on Learning Representations* (2020); https://openreview.net/forum?id=H1eA7AEtvS&utm_campaign=The.

[14] J. Deng, W. Dong, R. Socher, L. J. Li, K. Li, F. F. Li, Imagenet: A large-scale hierarchical image database, in *2009 IEEE Computer Society Conference on Computer Vision and Pattern Recognition* (IEEE, 2009), pp. 248–255.

[15] Z. Liu, P. Luo, X. Wang, X. Tang, Deep learning face attributes in the wild, in *IEEE International Conference on Computer Vision* (ICCV, 2015), pp. 3730–3738.

[16] Z. Liu, P. Luo, X. Wang, X. Tang, Deep learning face attributes in the wild, in *Proceedings of International Conference on Computer Vision (ICCV)* (December 2015).

[17] K. J. Holyoak, D. Simon, Bidirectional reasoning in decision making by constraint satisfaction. J. Exp. Psychol. Gen. 128, 3–31 (1999).

[18] A. Paullada, I. D. Raji, E. M. Bender, E. Denton, A. Hanna, Data and its (dis)contents: A survey of dataset development and use in machine learning research. Patterns 2, 100336 (2021). 34820643 10.1016/j.patter.2021.100336 PMC8600147

[19] C. Wei, X. Yang, in *2021 3rd International Conference on Electrical Engineering and Control Technologies (CEECT)* (IEEE, 2021), pp. 203–207.

[20] M. Labayen, R. Vea, J. Flórez, N. Aginako, B. Sierra, Online student authentication and proctoring system based on multimodal biometrics technology. IEEE Access 9, 72398–72411 (2021).

[21] F. Poursabzi-Sangdeh, D. G. Goldstein, J. M. Hofman, J. W. Wortman Vaughan, H. Wallach, Manipulating and measuring model interpretability, in *Proceedings of the 2021 CHI Conference on Human Factors in Computing Systems* (Association for Computing Machinery, 2021), pp. 1–52.

[22] G. Bansal, T. Wu, J. Zhou, R. Fok, B. Nushi, E. Kamar, M. T. Ribeiro, D. Weld, Does the whole exceed its parts? The effect of AI explanations on complementary team performance, in *Proceedings of the 2021 CHI Conference on Human Factors in Computing Systems* (Association for Computing Machinery, 2021), pp. 1–16.

[23] S. Gaube, H. Suresh, M. Raue, A. Merritt, S. J. Berkowitz, E. Lermer, J. F. Coughlin, J. V. Guttag, E. Colak, M. Ghassemi, Do as AI say: Susceptibility in deployment of clinical decision-aids. npj Digit. Med. 4, 31 (2021). 33608629 10.1038/s41746-021-00385-9 PMC7896064

[24] A. Storkey, When training and test sets are different: Characterizing learning transfer, in *Dataset Shift in Machine Learning* (MIT Press, 2009), vol. 30, p. 3.

[25] M. Mosbach, M. Andriushchenko, D. Klakow, *On the Stability of Fine-Tuning BERT: Misconceptions, Explanations, and Strong Baselines* (ICLR, 2020).

[26] J. Devlin, M.-W. Chang, K. Lee, K. Toutanova, BERT: Pre-training of deep bidirectional transformers for language understanding, in *Proceedings of the 2019 Conference of the North American Chapter of the Association for Computational Linguistics: Human Language Technologies* (Association for Computational Linguistics, 2019), vol. 1.

[27] G. Patrini, A. Rozza, A. Krishna Menon, R. Nock, L. Qu, Making deep neural networks robust to label noise: A loss correction approach, in *IEEE Conference on Computer Vision and Pattern Recognition* (CVPR, 2017), pp. 1944–1952.

[28] P. Skryjomski, B. Krawczyk, Influence of minority class instance types on SMOTE imbalanced data oversampling, in *First International Workshop on Learning with Imbalanced Domains: Theory and Applications LIDTA* (PMLR, 2017), pp. 7–21.

[29] Y. Liu, M. Ott, N. Goyal, J. Du, M. Joshi, D. Chen, O. Levy, M. Lewis, L. Zettlemoyer, V. Stoyanov, RoBERTa: A robustly optimized BERT pretraining approach. arXiv:1907.11692 [cs.CL] (26 July 2019).

[30] A. Williams, N. Nangia, S. Bowman, A broad-coverage challenge corpus for sentence understanding through inference, in *Proceedings of the 2018 Conference of the North American Chapter of the Association for Computational Linguistics: Human Language Technologies, Volume 1 (Long Papers)* (Association for Computational Linguistics, 2018), pp. 1112–1122.

[31] W. Dieterich, C. Mendoza, T. Brennan, “COMPAS risk scales: Demonstrating accuracy equity and predictive parity” (Technical Report , Northpointe Inc, 2016).

[32] N. Sae-Bae, X. Sun, H. T. Sencar, N. D. Memon, Towards automatic detection of child pornography, in *2014 IEEE International Conference on Image Processing (ICIP)* (IEEE, 2014), pp. 5332–5336.

[33] K. Harrison, SB 10: Punishment before conviction? Alleviating economic injustice in California with Bail Reform. Univ. Pac. Law Rev. 49, 533 (2017).

[34] N. W. State v Loomis; www.courts.ca.gov/documents/BTB24-2L-3.pdf.

[35] B. Martinez Neda, Y. Zeng, S. Gago-Masague, Using machine learning in admissions: Reducing human and algorithmic bias in the selection process, in *Proceedings of the 52nd ACM Technical Symposium on Computer Science Education* (ACM, 2021), pp. 1323–1323.

[36] R. Fogliato, A. Xiang, Z. Lipton, D. Nagin, A. Chouldechova, On the validity of arrest as a proxy for offense: Race and the likelihood of arrest for violent crimes, in *Proceedings of the 2021 AAAI/ACM Conference on AI, Ethics, and Society* (Association for Computing Machinery, 2021), pp. 100–111.

[37] A. Tversky, D. Kahneman, *Behavioral Decision Making* (Springer, 1985), pp. 25–41.

[38] I. P. Levin, G. J. Gaeth, How consumers are affected by the framing of attribute information before and after consuming the product. J. Consum. Res. 15, 374–378 (1988).

[39] E. Shafir, R. A. LeBoeuf, Rationality. Annu. Rev. Psychol. 53, 491–517 (2002). 11752494 10.1146/annurev.psych.53.100901.135213

[40] R. J. Simon, L. Mahan, Quantifying burdens of proof: A view from the bench, the jury, and the classroom. Law Soc. Rev. 5, 319–330 (1970).

[41] D. Simon, D. C. Krawczyk, K. J. Holyoak, Construction of preferences by constraint satisfaction. Psychol. Sci. 15, 331–336 (2004). 15102143 10.1111/j.0956-7976.2004.00678.x

[42] D. Simon, D. M. Stenstrom, S. J. Read, The coherence effect: Blending cold and hot cognitions. J. Pers. Soc. Psychol. 109, 369–394 (2015). 26167800 10.1037/pspa0000029

[43] Z. Jin, S. Levine, F. Gonzalez Adauto, O. Kamal, M. Sap, M. Sachan, R. Mihalcea, J. Tenenbaum, B. Schölkopf, When to make exceptions: Exploring language models as accounts of human moral judgment, in *Advances in Neural Information Processing Systems* (2022); https://proceedings.neurips.cc/paper_files/paper/2022/hash/b654d6150630a5ba5df7a55621390daf-Abstract-Conference.html.

[44] M. Forbes, J. D. Hwang, V. Shwartz, M. Sap, Y. Choi, Social chemistry 101: Learning to reason about social and moral norms, in *Proceedings of the 2020 Conference on Empirical Methods in Natural Language Processing* (EMNLP, 2020), pp. 653–670.

[45] A. Chouldechova, A. Roth, A snapshot of the frontiers of fairness in machine learning. Commun. ACM 63, 82–89 (2020).

[46] S. Barocas, A. Guo, E. Kamar, J. Krones, M. R. Morris, J. W. Vaughan, W. D. Wadsworth, H. Wallach, Designing disaggregated evaluations of AI systems: Choices, considerations, and tradeoffs, in *Proceedings of the 2021 AAAI/ACM Conference on AI, Ethics, and Society* (AIES, 2021), pp. 368–378.

[47] T. Gebru, J. Morgenstern, B. Vecchione, J. W. Vaughan, H. Wallach, H. Daumé III, K. Crawford, Datasheets for datasets. Commun. ACM 64, 86–92 (2021).

[48] M. Mitchell, S. Wu, A. Zaldivar, P. Barnes, L. Vasserman, B. Hutchinson, E. Spitzer, I. D. Raji, T. Gebru, Model cards for model reporting, in *Proceedings of the Conference on Fairness, Accountability, and Transparency* (Association for Computing Machinery, 2019), pp. 220–229.

[49] I. D. Raji, I. E. Kumar, A. Horowitz, A. Selbst, The fallacy of AI functionality, in *2022 ACM Conference on Fairness, Accountability, and Transparency* (Association for Computing Machinery, 2022), pp. 959–972.

[50] H. Adam, A. Balagopalan, E. Alsentzer, F. Christia, M. Ghassemi, Mitigating the impact of biased artificial intelligence in emergency decision-making. Commun. Med. 2, 149 (2022). 36414774 10.1038/s43856-022-00214-4 PMC9681767

[51] H. Chen, A. Gallagher, B. Girod, Describing clothing by semantic attributes, in *ECCV* (Springer, 2012), pp. 609–623.

[52] Z. Liu, P. Luo, S. Qiu, X. Wang, X. Tang, DeepFashion: Powering robust clothes recognition and retrieval with rich annotations, in *IEEE Conference on Computer Vision and Pattern Recognition* (CVPR, 2016).

[53] L. Bossard, M. Guillaumin, L. Van Gool, Food-101—Mining discriminative components with random forests, in *ECCV* (Springer, 2014).

[54] A. Singla, L. Yuan, T. Ebrahimi, Food/non-food image classification and food categorization using pre-trained GoogLeNet model, in *Proceedings of the 2nd International Workshop on Multimedia Assisted Dietary Management* (2016), pp. 3–11.

[55] I. Donadello, M. Dragoni, Ontology-driven food category classification in images, in *20th International Conference on Image Analysis and Processing* (ICIAP, 2019), vol. 11752, pp. 607–617.

[56] A. Khosla, N. Jayadevaprakash, B. Yao, F.-F. Li, Novel dataset for fine-grained image categorization: Stanford dogs, in *FGCV CVPR* (Citeseer, 2011), vol. 2.

[57] O. M. Parkhi, A. Vedaldi, A. Zisserman, C. V. Jawahar, in *IEEE CVPR* (IEEE, 2012), pp. 3498–3505.

[58] D. Borkan, L. Dixon, J. Sorensen, N. Thain, L. Vasserman, Nuanced metrics for measuring unintended bias with real data for text classification. WebConf. 2, 491–500 (2019).

[59] L. Hanu, Unitary team, Detoxify, Github (2020); https://github.com/unitaryai/detoxify.

[60] G. E. Hinton, N. Srivastava, A. Krizhevsky, I. Sutskever, R. R. Salakhutdinov, Improving neural networks by preventing co-adaptation of feature detectors. arXiv:1207.0580 [cs.NE] (3 July 2012).

[61] A. L. Maas, R. E. Daly, P. T. Pham, D. Huang, A. Y. Ng, C. Potts, Learning word vectors for sentiment analysis, in *Proceedings of the 49th Annual Meeting of the Association for Computational Linguistics: Human Language Technologies-Volume 1* (ACL, 2011), pp. 142–150.

[62] C. Schuhmann, R. Kaczmarczyk, A. Komatsuzaki, A. Katta, R. Vencu, R. Beaumont, J. Jitsev, T. Coombes, C. Mullis, LAION-400M: Open dataset of CLIP-filtered 400 million image-text pairs, in *NeurIPS Workshop Datacentric AI*, no. FZJ-2022-00923 (Jülich Supercomputing Center, 2021).

[63] F. Dernoncourt, J. Y. Lee, PubMed 200k RCT: A dataset for sequential sentence classification in medical abstracts, in Proceedings of the Eighth International Joint Conference on Natural Language Processing (Volume 2: Short Papers) (Asian Federation of Natural Language Processing, 2017), p. 308.

[64] O.-M. Camburu, T. Rocktäschel, T. Lukasiewicz, P. Blunsom, e-SNLI: Natural language inference with natural language explanations, in *Advances in Neural Information Processing Systems* (2018).

