# Supplementary material for: Judging facts, judging norms: Training machine learning models to judge humans requires a modified approach to labeling data
Source: Sci Adv. 2023 May 10;9(19):eabq0701. doi: 10.1126/sciadv.abq0701 (PMC10171805; doi:10.1126/sciadv.abq0701)
Supplement: Supplementary file 1 — Supplementary Text Tables S1 to S8 Legends for data S1 to S12 Figs. S1 to S4 [file sciadv.abq0701_sm.pdf]

Science Advances
AAAS

Supplementary Materials for

Judging facts, judging norms: Training machine learning models to judge humans requires a modified approach to labeling data

Aparna Balagopalan *et al.*

Corresponding author: Aparna Balagopalan, aparnab@mit.edu

Sci. Adv. **9**, eabq0701 (2023)
DOI: 10.1126/sciadv.abq0701

The PDF file includes:

Supplementary Text
Tables S1 to S8
Legends for data S1 to S12
Figs. S1 to S4

Other Supplementary Material for this manuscript includes the following:

Data S1 to S12

A Supplementary Text

A.1 Annotator Label Quality

We took several steps to ensure high data quality: (1) placing restrictions on worker qualifications, (2) releasing data in batches to allow higher quality control and allowing for detailed manual review before accepting jobs, (3) adding attention checks to two image datasets, and "gold-standard" questions to the text dataset, as described below.

We also added a sentence saying: "We will use an algorithm to ensure that Turkers who submit random/repetitive answers will not be compensated" within every HIT to alert the MT participants to pay attention.

The attention check for the image datasets consisted of a large text block, with the following text:

> Understanding human reasoning for everyday decisions is very important, so we thank you for your contributions. We are interested in knowing certain factors about you, the decision-maker. Since our experiments rely strongly on the change of instructions, we want to ensure that you consider all directions closely. To demonstrate this, please ignore the color options below, and select the option "other". Then, explain your choice by writing the single digit NUMBER in the empty box below the option "other". Answering the material in this question provides useful information, since having more data about participants is helpful for conducting the experiment. Thank you for entering the requested information.

Below this text instruction three radio buttons could be selected, the last of which was "other". The portion marked as NUMBER in the text above was replaced with a random number between zero and nine for each HIT, which the participants entered in a text box below the last radio button. We accepted all jobs where this number was entered correctly.

For the text dataset, we added in “gold-standard questions”, that is, questions of the same form as the objects to be labeled, but with unambiguously correct answers. For example, the comment “The sky is blue.” : this does not violate the guideline, nor does it contain any of the three factual features in the text Comment policy. These questions appeared either as the first or last question in each HIT.

We manually reviewed the attention check answers using the MT requester review interface, and we accepted the jobs if they correctly answered the check. Only jobs that are finally in approved status are used in our analyses. We also defined a proxy for data quality as the proportion of correctly completed attention checks (e.g., filled in the correct digit) in the accepted jobs, which we estimated to be higher than 99.3% for the three datasets with quality checks (Meal, Pet, Comment).

The layout for the Clothing dataset collection HITs was implemented differently from the other three datasets. Each HIT contained one object to be labeled (instead of randomly grouping five together as shown in Figure S1 for the other three datasets). Due to the brevity of the Clothing dataset HITs, we implemented a proxy for the attention check using the completion time. The threshold for completion time was set to be 1 second to filter out bots rather than speeders . However, no rejections were handed out due to this criteria.

Additionally, we obtained a few data-points in the normative condition wherein participants answered that the rule/code is not violated, but checked off that a specific feature (e.g., a large-sized dog) was present (or vice versa). In most cases, we rejected jobs where such behavior was consistently observed (since it indicated that instructions were misunderstood, and hence fail an implicit attention check), but retained jobs where such behavior was shown as one-off cases by participants with a high approval rate for past jobs. The rates of such data points are consistently low in the final datasets. We removed these datapoints as a data cleaning step before all analyses.

A.1.1 Pilot Studies

We conducted pilot studies before launching the main study to ensure that labels collected were reliable with 20 participants labeling each object in each dataset. In these pilots, we also experimented with minor variations to the task prompts. For example, in a pilot study for the Pet dataset, we observed that asking participants to decide if 'dogs were suitable for apartment living' in the descriptive with context condition tended to produce results more similar to the normative condition (as the word 'suitable' is value-laden and could indirectly evoke concepts like apartment pet codes in the annotator's mind). We modified this to say 'would not be out of place in a small living space or apartment' in the main study. For the normative condition in the Meal dataset, we experimented with slightly changing the prompt by removing the sentence 'Why or why not. Give ALL reasons that would justify your decision that the restrictions are or are not violated' in the normative condition below each image to reduce text added in the normative condition in comparison to the descriptive condition (i.e., just specifying in the main task instructions that the rule violation judgment was to be made, and all possible justifications checked off) in a pilot. For the Comment dataset, we modified a factual feature from 'Contains negative comments or slurs against race, sexual orientation or gender' to 'Contains negative comments about race, sexual orientation or gender' based on worker feedback during pilots.

These pilot studies were also used to inform the amount paid to participants per HIT (where we aimed to pay participants $12/hour) using statistics of HIT completion time. Note that we do not include data from these pilot studies in any analyses, and participants who participated in pilots and provided high-quality labels were allowed to participate in the main experiments.

Despite these steps we took to ensure good quality and conform to high standards for data collection, we acknowledge two minor typographical errors in the data collection prompts that propagated to the main study: in the normative pet code prompts for some micro-tasks on MT "aggressive" was spelled incorrectly as "aggresive" in a few spots, and in the descriptive

condition prompts for the Meal dataset, “image” was used ungrammatically instead of “images” in the phrase “meals depicted in the images below have” in one micro-task batch out of ten (while there were multiple images to label per HIT/micro-task, which the rest of the instructions clearly described). For the Meal descriptive batch, we verified that this did not cause labels to vary significantly (Kruskal Wallis test; $p = 0.3$) by re-running this batch after the full study was completed, and hence retain the original batch in the main dataset. We also received no complaints/questions from MT participants, so conclude this had no effect.

A.2 Details of Data Curation and Selection

For the Clothing dataset, we ran a pilot attribute labeling study for 4000 images with the instruction “Answer yes/no questions about some relevant attributes of the clothing shown” for obtaining noisy labels for our three factual features. These labels were then used to sample 2000 images for labeling. For the Meal and Pet datasets, we explicitly tried to sample more contentious or ambiguous violations of the code/policy since this is where we expect to see the most difference between labeling conditions. We also included objects with clear violations and non-violations of the code/policy for the purpose of training ML models, and hence maintaining balance in classes. Note that the size/aspect ratio of the images often varied in the Meal and Pet datasets—both within and across datasets the objects were sampled from. Additionally, we attempted to ensure a diverse collection of images, since diversity of images would be a factor that influences differences between conditions. Note, however, while we checked that no exact duplicates were present in the dataset, perceptually similar images/text samples do exist (as they do in the original datasets the objects were sampled from). We note that there are a few images sampled from “Pixaby” in the Meal dataset that have identical visual content but differing aspect ratios or image hashes. We estimate the proportion of this to $\approx 0.45\%$ of the full dataset (i.e., ≤ 9 duplicate images).

For the Comment dataset, we used the labels indicating toxicity of a comment present in the CivilComments (*56*) dataset (using their definition of "toxicity", identity attacks, obscenity, insults, and threats) to perform a more controlled and precise sampling. We first filtered the dataset by removing long comments. Then, we selected 70% text samples for which the comment – each with continuous toxicity and factual feature labels between 0 to 1 – had high contentiousness (following the definition set a priori), and 30% low contentiousness. In each of these cases, we ensured that an equal proportion of each factual feature was present in our dataset (e.g., $\approx 33\%$ text samples using obscene language). We also tried to ensure that labels covered the full range of possible factual feature values (0 to 1) in order to build a balanced dataset. This required adjusting the sampling procedure throughout data collection. In all cases, we shuffled the order of the objects randomly after sampling to avoid any determinism in order.

A.3 Difference between Descriptive and Normative

We find strong evidence to support the hypothesis that the judgment labels generated by the descriptive and normative conditions are not drawn from the same distribution in all four settings (Dress: $p = 1.61 \times 10^{-07}$; Meal: $p = 3.35 \times 10^{-74}$; Pet: $p = 1.87 \times 10^{-87}$; Comment: $p = 1.33 \times 10^{-12}$ with a Kruskal-Wallis H-test). Further, this difference also has a consistent direction across the four datasets: for each setting, the average descriptive label for an object is significantly higher than its corresponding average normative label. (Dress: $p = 3.35 \times 10^{-39}$; Meal: $p = 4.36 \times 10^{-244}$; Pet: $p = 1.46 \times 10^{-291}$; Comment: $p = 2.27 \times 10^{-95}$ with a one-sided Wilcoxon signed-rank test). Note that these results all survive the Bonferroni correction (*65*) for multiple testing, and remain statistically significant. Significant differences are also observed for the objects with high contentiousness.

A.4 Difference between Descriptive-with-context and Normative

A Kruskal-Wallis H-test indicates that the judgment labels generated by the descriptive-with-context and normative conditions are not drawn from the same distribution in all four settings (Dress: $p = 5.22 \times 10^{-29}$; Meal: $p = 1.14 \times 10^{-59}$; Pet: $p = 6.68 \times 10^{-71}$; Comment: $p = 1.36 \times 10^{-27}$).

A.5 Difference between Descriptive and Descriptive-with-context

We find that the effect of context is dataset-dependant when comparing descriptive and descriptive-with-context labels: the descriptive and descriptive-with-context labeling procedures are significantly different on average for two of the four settings (Clothing: $p = 5.55 \times 10^{-11}$; and Comment: $p = 8.15 \times 10^{-05}$ dataset with a Kruskal-Wallis H-test). For the other two settings (Meal and Pet), the descriptive and descriptive-with-context settings are *not* significantly different on average (Meal: $p = 0.22$ and Pet: $p = 0.08$).

Further, on computing individual tests in Table S1, we find that the proportion of objects labeled significantly differently is consistently low (from 3.25-4.15% across all objects, and between 3.89-9.28% for objects with high contentiousness).

Dataset	Overall	High Contention	Low Contention
Clothing	3.25%	9.28%	1.15%
Meal	3.70%	6.95%	1.64%
Pet	3.65%	4.76%	2.57%
Comment	4.15%	3.89%	4.75%

Table S1: Comparing descriptive and descriptive-with-context labels, we see that proportions of objects significantly different is low.

A.6 Impact of Threshold for Assigning a Binary Label

We find that differences between descriptive and normative condition persist across a range of thresholds as seen in Figure S2.

A.7 Effect of Demographics

A.7.1 Testing for Group Differences

Across all datasets, we find that median gender and age composition of participants is not significantly different between descriptive and normative labelers, except for gender in the Clothing dataset ($p = 0.004$), and gender in the Meal dataset ($p = 0.006$) with a Kruskal-Wallis H-test. Note that the difference for Clothing dataset could be artificially driven by missing information, since gender information is available for only 140 descriptive labelers, and 260 normative labelers. Further, we show below that even after controlling for age and gender, the descriptive vs. normative label differences are strongly retained at $p < 0.001$ in the Meal dataset.

A.7.2 Mixed Effect Modelling with Demographic Effects

We also analyzed data with mixed-effects models controlling for self-reported participant gender (male, female, other). We used a linear mixed effect model to analyze the relationship between the average judgment label of objects and the condition assigned (descriptive vs. normative) and median gender and median age group of participants labeling each object. This specification included random intercepts for each object. We perform this analysis on two datasets: Meal (characterized by lowest uniformity in demographics of participants assigned to different conditions) and Comment (characterized by highest uniformity in demographics of participants assigned to different conditions). In Table S2, we observe that even after controlling for age and gender, group or condition assignment significantly affects the assigned label at $p < 0.001$ and has high effects.

	Meal				Comment			
Variable	Coefficient	SE	z	p	Coefficient	SE	z	p
Group	-0.211	0.006	-37.461	<0.001	-0.067	0.003	-22.523	<0.001
Gender	0.006	0.007	0.884	0.376	-0.018	0.007	-2.661	0.008
Age	0.023	0.011	2.020	0.043	-0.087	0.021	-4.152	<0.001

Table S2: Linear mixed-regression models for assigned judgment label (with fixed covariates including median age and median gender of participants for each object). Coefficient: regression coefficient; SE: standard error; z:t-value; p:probability of committing a Type I error.

A.8 Robustness Checks for Data

Our study design is of a series of online controlled experimentation with independent recruitment for each dataset or task – i.e., recruitment for each dataset (Clothing, Meal, Pet, Comment) was performed independently, with participants assigned to a single condition within a dataset. With this design, participants may be allocated to different conditions in different datasets. Note that the participants are not informed of the condition they are assigned to, and the HITs are all designed as data labeling jobs (as routinely seen on MT). Due the varied domain of tasks as well as differences in instructions and format of HITs (e.g., Clothing vs Pet had different instructions, format, as well as number of images per HIT), we do not expect such cross-dataset overlaps to affect our results. Additionally, for the Clothing dataset some intermediate pilot experiments utilized a within-subject design (where participants saw multiple conditions within a given dataset), and we found similar trends in results. Nevertheless, we attempted to avoid and minimize such cross-dataset overlaps by collecting datasets sequentially, and setting participant qualifications appropriately. However, since data collection was performed by two members of the research team (2 datasets each) over a 2 year time-frame, and MT requires a unique requester account for creating/submitting jobs, some cross-dataset overlaps were unavoidable in a few cases. As a robustness check for our findings, we repeated our data analysis after full data

collection by removing such overlaps that occur within 2 months (or 60 days) of each other. Similar to the results shown in the main text, we find strong evidence to support the hypothesis that the labels are different under descriptive and normative conditions (Dress: p = 2.67e-07; Meal: p = 1.32e-70; Pet: p = 1.58e-96; Comment: p = 1.12e-38 with a Kruskal-Wallis H-test). In the Table below, we show our results from Figure 3 after removing such overlaps as a robustness check – we observe that significant differences between the descriptive and normative, as well as normative and descriptive-with-context persists in between 6-53% of the contentious objects.

As seen in Table S3: removing cross-task overlaps between studies - the dataset size decreases in some cases, due to which the proportion of images having significantly different labels between conditions is reduced from as computed before removing such overlaps. However, the key observation that over 5% images have significantly different labels between conditions is strongly retained.

**Dataset**	**Normative-Descriptive**		**Normative-Descriptive-with-Context**	
	**High Contention**	**Low Contention**	**High Contention**	**Low Contention**
Dress	13.59%	1.95%	14.76%	3.23%
Meal	53.10%	18.43%	49.66%	15.37%
Pet	29.79%	22.69%	23.86%	18.24%
Comments	5.78%	6.59%	13.29%	6.74%

Table S3: Differences between labels: robustness check

Note that in a few initial iterations of the Clothing data collection, some experiments utilized a within-subject design (where participants saw multiple conditions), and we found similar trends in results. We removed these from the final Clothing dataset. A small proportion of these participants may have then participated in the labeling tasks for the other three settings (i.e., Meal, Pet, Comment), but such re-participation may have occurred only after a five/six-month

time period (given that data collection was sequential, with a difference of five-six months between data collection ending for Clothing, and collection starting for the other three settings). Further, we estimate the proportion of such participation to be very low given the variation in the MT worker pool over time ($\leq 3\%$).

A.8.1 Robustness Check: Framing Normative Tasks as Violations vs Compliance

We find no significant difference between normative labels collected under a framing of "violations" (more negative) or "compliance" framing (more positive) for all four datasets on a representative subsample. The subsampling procedure involved selecting 200 objects from each of the four datasets such that the overall distributions of the judgment, as well as factual features, were not statistically different from that of the whole dataset. On this sample, statistical testing comparing the average judgment label for objects under the "violation" and "compliance" framing leads to the following results with a Kruskal Wallis H-test: Clothing (p-value=0.147), Meal (p-value=0.908), Pet (p-value=0.585), Comment (p-value=0.786). In comparison, testing differences between descriptive and normative framing with "violations" on the same subsample leads to the following results: Clothing (p-value=0.135), Meal (p-value$<$0.001), Pet (p-value$<$0.001), Comment (p-value=0.056). Hence, we conclude that positive-negative framing effects are low in our study. However, we emphasize that our current sample size was powered to detect group effects $\geq$0.2, and more work investigating the effect of positive vs negative framing across different contexts (e.g., medical decisions) might reveal interesting trends.

A.8.2 Robustness Check: Violation Question Order

We conducted a robustness check testing whether the difference we observe is driven by coherence effects and avoidance of cognitive dissonance between assessment of factual findings and the violation judgment. Specifically, we tested whether the order of asking the violation judgment: i.e., asking "Does this object violate the norm? Justify by choosing factual features."

vs. "Decide if any factual features justifying violation are present. Does this object violate the norm?" produces different labels for a representative sample of 200 objects for each of the four settings (i.e., similar to the previous robustness check). We observed that changing the order in this manner produces labels not significantly different ($p \geq 0.05$) in three out of the four settings: Clothing (p-value=0.993), Meal (p-value=0.170), and Pet (p-value=0.096). For the Comment dataset, the treatment resulted in significantly different labels (p=0.019). However, on Bonferroni correction for multiple testing, this change is no longer significant. From this, we conclude that we passed the robustness check but there is some evidence that ordering might matter for the Comment dataset.

A.9 Machine Learning Model: Training and Evaluation Details

An illustration of our model training and evaluation setup is in the Figure S3.

The final hyperparameter settings for all models are shown in Table S4.

Dataset	Descriptive	Normative
Dress	$\{0.01, 128, 0.5\}$	$\{0.1, 128, 0.5\}$
Meal	$\{0.01, 32, 0.5\}$	$\{0.01, 32, 0.1\}$
Pet	$\{0.01, 64, 0.5\}$	$\{0.001, 128, 0.5\}$

Table S4: Hyperparameter settings for all ResNet50 models for each dataset (identified after grid-search hyperparameter tuning based on validation performance). The values shown in each cell is {learning rate, batch size, gamma for weight decay}. Note that these hyperparameters are also used for the data noising and dataset size ablation studies.

To compute test accuracy/F1-scores for both image and text datasets, we first obtain a single test label for each object in the test set using a specific threshold of inter-annotator agreement. For the main results shown in the paper, we use a majority vote or a threshold of 50% following most of prior literature. In addition, we use a threshold of 0.5 for computing all threshold-dependent metrics of performance unless specified otherwise. We also verified that reporting

Contention	Train: Descriptive, test: Normative	Train: Normative, test: Normative	Dataset
High	0.669 ± 0.0176	0.7283 ± 0.0186	Clothing
Low	0.9674 ± 0.0029	0.974 ± 0.0048	Clothing
All	0.8952 ± 0.0062	0.9145 ± 0.0051	Clothing
High	0.5725 ± 0.0192	0.7017 ± 0.016	Meal
Low	0.8056 ± 0.0358	0.935 ± 0.0061	Meal
All	0.7123 ± 0.0282	0.8417 ± 0.0093	Meal
High	0.6037 ± 0.0093	0.6794 ± 0.007	Pet
Low	0.7445 ± 0.0346	0.9066 ± 0.0131	Pet
All	0.6681 ± 0.0144	0.7833 ± 0.0095	Pet
High	0.6576 ± 0.02	0.7218 ± 0.0218	Comment
Low	0.8859 ± 0.0191	0.9225 ± 0.0192	Comment
All	0.7304 ± 0.0154	0.7858 ± 0.0135	Comment

Table S5: Threshold dependent: Accuracy @ threshold = 0.5

performance on all test objects without aggregation on the test set (i.e., without assigning a single label per test object with majority vote) led to numerically similar results and trends. Tables S5 and S6 contain detailed performance metrics for all models. For the text dataset, we observed that the variance of performance between runs is relatively high. The F1-score of models trained on descriptive labels is substantially lower than models trained on normative label ($p < 0.05$ with Kruskal Wallis H-test), but has high variance. Note that all scripts were run on a shared compute cluster, so some were re-run if preempted by other jobs (random seeds were set in all cases to ensure reproducibility). We verified that the main trends (e.g., relative performance of the two text base models, larger impact of using normative data in comparison to model architecture changes, etc.) still hold across runs on different compute setups. The performance in predicting factual features is also generally high (over 70% accuracy/F1-score), except in a few cases with highly-imbalanced factual feature distributions.

From the results, as expected, the test performance on objects with low (normative) contentiousness is higher than that for objects with high (normative) contentiousness for metrics such as accuracy and F1.

Contention	Train: descriptive, test: normative	Train: normative, test: normative	dataset
High	0.6263 ± 0.025	0.7096 ± 0.0184	Clothing
Low	0.9664 ± 0.0029	0.9731 ± 0.005	Clothing
All	0.8949 ± 0.0062	0.914 ± 0.0052	Clothing
High	0.4687 ± 0.0483	0.7002 ± 0.0166	Meal
Low	0.753 ± 0.0581	0.9302 ± 0.0069	Meal
All	0.6384 ± 0.0521	0.8361 ± 0.0104	Meal
High	0.5391 ± 0.018	0.6784 ± 0.0064	Pet
Low	0.738 ± 0.0324	0.893 ± 0.0152	Pet
All	0.662 ± 0.0173	0.7791 ± 0.0106	Pet
High	0.6492 ± 0.0183	0.6917 ± 0.0163	Comment
Low	0.8546 ± 0.0214	0.8913 ± 0.0271	Comment
All	0.712 ± 0.013	0.7489 ± 0.0099	Comment

Table S6: Threshold dependent: F1-score @ threshold = 0.5

Figure S4 shows the model performance averaged across objects with high normative contentiousness. We observe similar trends as reported in the main text, though the magnitudes of differences are larger, and depend on the proportion of objects with high contentiousness in a dataset.

Note that the dataset sizes we used in this experiment are much smaller than what would be used in a realistic industrial application. We would expect that as dataset size and computational power increases, the performance of the descriptive and normative models would approach 100% accuracy on their respective labels. In the limit (with an infinite amount of data), we would expect the outputs of the descriptively (normatively) trained model to match the descriptive (normative) labels exactly; meaning that the accuracy loss from using descriptive labels to train a model to make normative judgments should then be equal to the proportion of the time that descriptive and normative labels disagree in our training set.

We also analysed a threshold-independent metric of AUPRC (Table S7; AUC was not used given high degree of class imbalance). Note that the hyperparameters are still chosen based on highest validation F1-macro (i.e., those in experiments above, and not tuned separately). For all

Contention	Train: descriptive, test: normative	Train: normative, test: normative	dataset
High	0.8283 ± 0.0126	0.8851 ± 0.0147	Clothing
Low	0.9918 ± 0.0007	0.9924 ± 0.001	Clothing
All	0.9612 ± 0.002	0.9698 ± 0.0017	Clothing
High	0.7023 ± 0.0123	0.7752 ± 0.01	Meal
Low	0.9802 ± 0.0021	0.9905 ± 0.0011	Meal
All	0.9194 ± 0.0046	0.9477 ± 0.0018	Meal
High	0.7542 ± 0.0151	0.7855 ± 0.0058	Pet
Low	0.9135 ± 0.0109	0.9396 ± 0.0054	Pet
All	0.8161 ± 0.0109	0.8485 ± 0.0051	Pet
High	0.6166 ± 0.0452	0.683 ± 0.0306	Comment
Low	0.9322 ± 0.0097	0.9337 ± 0.0209	Comment
All	0.7204 ± 0.0298	0.7591 ± 0.0189	Comment

Table S7: Threshold independent: AUPRC

four datasets (Clothing, Meal, Pet, Comment), we observe that AUPRC follows similar trends to accuracy: AUPRC for the descriptively-trained model is significantly lower ($p \leq 0.05$; Kruskal Wallis test) than that of the normatively-trained model in each case. We note the descriptive-normative label measurement error is not just that of miscalibration, as could be indicated by similar AUPRC, but significantly different accuracy: modifying thresholds for classification (i.e., for descriptive and normative models separately) in the limit of more data would not solve the issue, as evidenced by the high spread of data in Figure 4.

Note that for the data noising experiments, we randomly flip labels for a subset of the train and validation data points (and not all 20 labels for the dataset objects). To avoid test-set reuse, we use the same optimization hyperparameters for all noising and subsampling experiments. Further, as a robustness check, we verified that modifying optimization hyperparameters for these experiments do not cause the reported numbers to vary substantially. The only variations observed were in the Clothing dataset subsampling experiment (where loss in accuracy is comparable to 40-70% size reduction depending on optimization hyperparameters).

Metric	Val-descriptive	Val-normative	Dataset
Accuracy	0.8635	0.8573	Clothing
	0.8151	0.8061	Meal
	0.7355	0.7186	Pet
	0.6644	0.6637	Comment
AUC	0.9235	0.9193	Clothing
	0.8062	0.8669	Meal
	0.7871	0.7726	Pet
	0.7268	0.7125	Comment
F1-score	0.8628	0.8543	Clothing
	0.6950	0.8011	Meal
	0.7116	0.7096	Pet
	0.6634	0.6460	Comment

Table S8: Robustness checks for ML models: Ascertaining that the validation performance of models are comparable (particularly in terms of accuracy)

A.10 Robustness Checks for ML Modelling

We ensure that all descriptive and normative models have similar performance on the validation set, or that their optimization is performed to a similar degree (see Table S8). Note that the descriptive or normative modelling tasks might differ in difficulty, given the different distribution of rule violation labels, and factual feature labels/associations.

A.11 Impact of Measurement Error on Large External Benchmark

In the Comment case, we also tested how models trained on descriptive and normative labels perform on a closely-related large pre-existing benchmark. We tested the models on the CivilComments (*56*) test set which consists of approximately 100k text snippets, all retrieved from archives of the CivilComments discussion platform; note that the text for our Comments dataset is sampled from the CivilComments training set. A private company, Jigsaw (`https://jigsaw.google.com/`), collected labels for this data to identify toxic conver-

sational attributes. They elicited a label for each comment by asking labelers to “Rate the toxicity of this comment”.

We obtain violation predictions for each datapoint in the dataset, from our models trained on our descriptive and normative Comment datasets respectively, and compute the difference in our models' positive rates - how frequently they predict that an object is a violation. We again find that our descriptively-trained model outputs more “violation” classifications on this large test set (10.25%) than the normatively-trained model does (5.72%). By comparison, the original Civil Comments labels (which use prompts about specific levels of toxicity namely:(a) Very Toxic (a very hateful, aggressive, or disrespectful comment that is very likely to make you leave a discussion or give up on sharing your perspective), (b) Toxic (a rude, disrespectful, or unreasonable comment that is somewhat likely to make you leave a discussion or give up on sharing your perspective),(c) Hard to Say, (d) Not Toxic show about 5.95% of the posts as “toxic” (thresholding by considering a comment to be toxic if greater than 50% annotators label it so).

A.12 Impact of Measurement Error Could Vary by Subgroup

We analyse the error rate of the descriptive-model when compared to the normative labels for the Comment dataset. We particularly study the error rates for particular mentions – where mentions are annotated by humans, and are a part of the CivilComments dataset. For example, the classification accuracy for comments with “male” mentions is 48.26% (vs 54.78% with normative model), while that with “female” mentions is 57.88% (vs 64.55% with normative model). We also observe that predicted rate of online forum rule violations (i.e. positive-rate) for comments with male mentions is 75.22% vs 61.74% with descriptive model and normative models, in comparison to 76.06% vs 61.52% for comments with female mentions (all using ALBERT model as base). More work targeting differences in performance for other group

identities such as religion with samples adequately powered to test such effects is important. Such variations may be important to consider, in addition to research on algorithmic approaches to fairness (*45*) in AI systems. Importantly, the variations between descriptive and normative labeling we see may be influenced by social bias in data labeling (*46*).

A.13 Data Captions

Data S1. Descriptive labels for the Clothing dataset for all 2000 image objects.

Data S2. Normative labels for the Clothing dataset for all 2000 image objects.

Data S3. Descriptive-with-Context labels for the Clothing dataset for all 2000 image objects.

Data S4. Descriptive labels for the Meal dataset for all 2000 image objects.

Data S5. Normative labels for the Meal dataset for all 2000 image objects.

Data S6. Descriptive-with-Context labels for the Meal dataset for all 2000 image objects.

Data S7. Descriptive labels for the Pet dataset for all 2000 image objects.

Data S8. Normative labels for the Pet dataset for all 2000 image objects.

Data S9. Descriptive-with-Context labels for the Pet dataset for all 2000 image objects.

Data S10. Descriptive labels for the Comment dataset for all 2000 text objects.

Data S11. Normative labels for the Comment dataset for all 2000 text objects.

Data S12. Descriptive-with-Context labels for the Comment dataset for all 2000 text objects.

Identify if ANY of the following are true for the text sample in the box above:

Contains negative comments about race, sexual orientation, gender, religion, or other sensitive personal characteristics

○ Yes ○ No

Is threatening to a person, group, or organization

○ Yes ○ No

Uses obscene language

○ Yes ○ No

(A) Descriptive Prompt

Online Discussion Forum Guidelines: A post is acceptable <u>unless</u> it:

- Contains negative comments about race, sexual orientation, gender, religion, or other sensitive personal characteristics
- Is threatening to a person, group, or organization
- Uses obscene language

Are the discussion forum guidelines violated for the text sample in the box above?

○ Yes ○ No

Why or why not? Give ALL reasons that would justify your decision that the guidelines are or are not violated

Contains negative comments about race, sexual orientation, gender, religion, or other sensitive personal characteristics

○ Yes ○ No

Is threatening to a person, group, or organization

○ Yes ○ No

Uses obscene language

○ Yes ○ No

(B) Normative Prompt

Identify if the text sample in the box above is respectful by determining if ANY of the following are true:

Contains negative comments about race, sexual orientation, gender, religion, or other sensitive personal characteristics

○ Yes ○ No

Is threatening to a person, group, or organization

○ Yes ○ No

Uses obscene language

○ Yes ○ No

(C) Descriptive-with-context Prompt

Figure S1: Screenshots of prompts used for descriptive and normative settings in the Comment dataset.

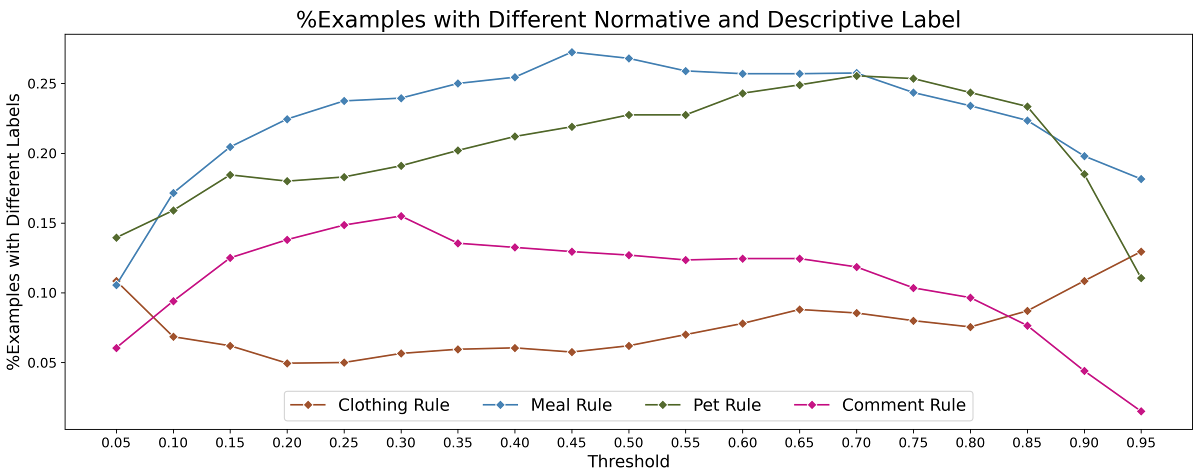

Figure S2: %Objects classified as violations are different between descriptive and normative conditions across a range of thresholds

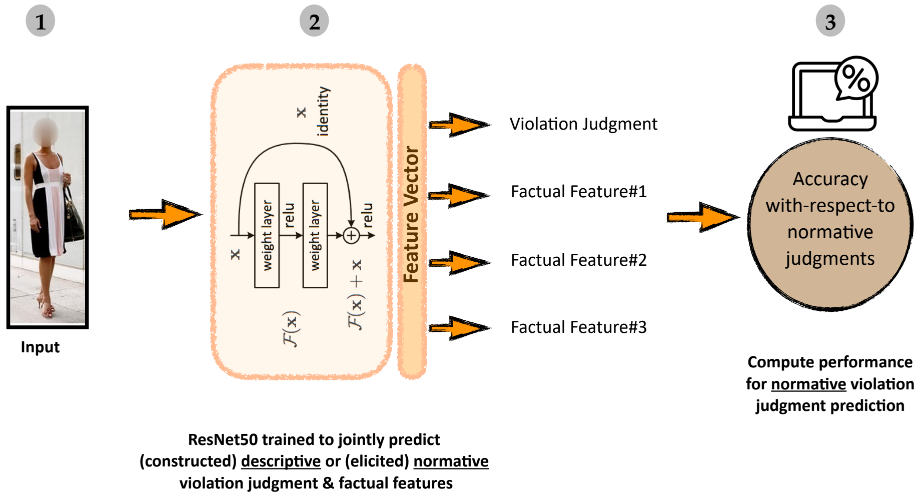

Figure S3: Model Training/Testing Setup: Models are trained to jointly predict the rule violation label, as well as 3 factual features/predicates for each dataset.

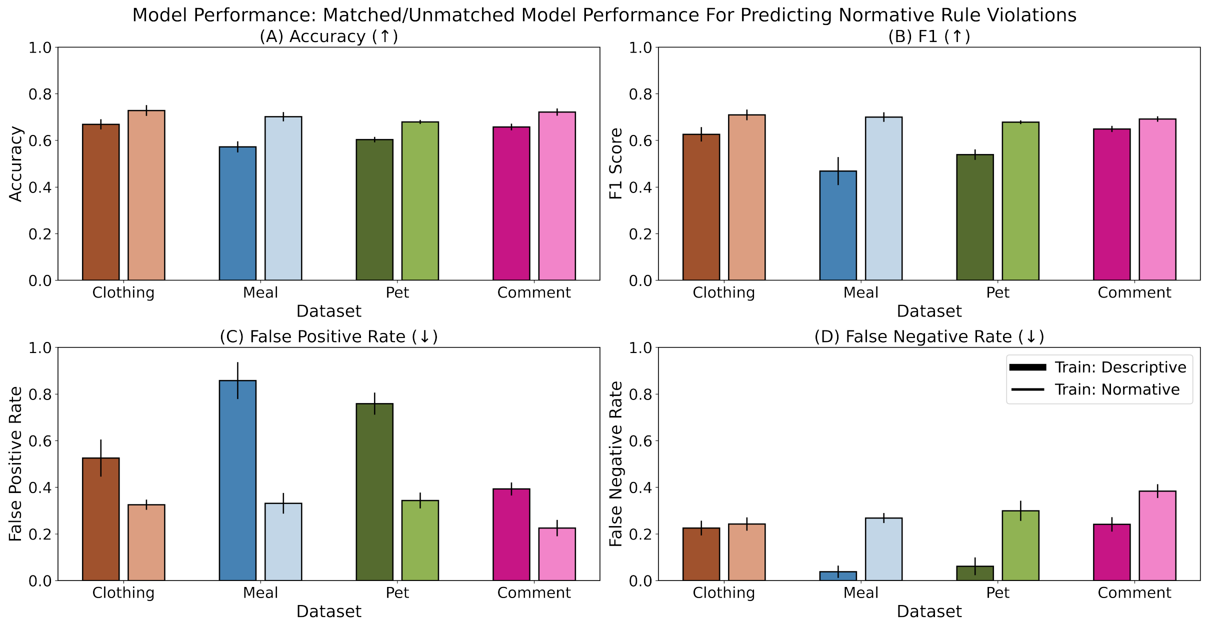

Figure S4: Model performance differences on high contention objects – those with normative labels close to 0.5 – are often higher.
